# Traits and functions of alpine plant communities respond strongly but not always sufficiently to *in situ* climate change

**DOI:** 10.1111/nph.70503

**Published:** 2025-08-29

**Authors:** Billur Bektaş, Gemma Rutten, Amélie Saillard, Rodrigue Friaud, Cindy Arnoldi, Julien Renaud, Maya Guéguen, Arnaud Foulquier, Jérôme Poulenard, Emilie Lyautey, Jean‐Christophe Clément, Wilfried Thuiller, Tamara Münkemüller

**Affiliations:** ^1^ Univ. Grenoble Alpes, Univ. Savoie Mont Blanc, CNRS, LECA F‐38000 Grenoble France; ^2^ Department of Environmental Systems Science, Institute of Integrative Biology ETH Zürich 8092 Zürich Switzerland; ^3^ Institute of Plant Sciences University of Bern CH‐3013 Bern Switzerland; ^4^ Departement of Biology, École Normale Supérieure PSL University 75005 Paris France; ^5^ Univ. Savoie Mont‐Blanc, Univ. Grenoble Alpes, CNRS, EDYTEM F‐73000 Chambéry France; ^6^ University Savoie Mont Blanc, INRAE, CARRTEL 74200 Thonon‐Les‐Bains France

**Keywords:** alpine plants, climate change, ecosystem functions, leaf–root coordination, mycorrhizal colonization, plant economics space, Plant functional traits, transplant experiment

## Abstract

- Increasing climate warming and summer droughts are known to affect mountain plant communities, their functional traits and life strategies. However, little is known about how strongly and efficiently communities respond to climate change, and how tightly plant responses are linked to responses of ecosystem functions.
- To test this, we transplanted alpine plant communities to subalpine conditions, exposing them to warming and drying. We compared these transplanted communities to alpine and subalpine control communities to assess their responses.
- Five years after transplantation, we found slower growth (e.g. lower leaf nitrogen) and more outsourcing strategies (e.g. lower specific root length) in the warmer and drier subalpine control communities compared to the alpine controls, probably due to drought. Traits of warmed alpine communities shifted toward subalpine controls. However, neither below‐ nor aboveground traits nor productivity of plants fully acclimated to subalpine conditions. Nevertheless, standard litter decomposition rates, arbuscular colonization and bacterial biomass showed no acclimation lag to the subalpine controls.
- Significant but insufficient acclimation of plant functional traits and strategies is prone to result in maladapted plant productivity, impairing competitiveness with better adapted subalpine species and leading to the temporally delayed loss of ecosystem features specific to alpine environments.

## Abstract 2

See also the Commentary on this article by Andraczek, **249**: 1089–1091.

## Introduction

Plant functional traits are essential predictors of plant performance (Soudzilovskaia *et al*., 2013; Adler *et al*., 2014; Falster *et al*., 2018) and ecosystem functioning under climate change (Violle *et al*., 2007). Trait‐based approaches have allowed identifying trade‐offs that underpin ecological strategies in large‐scale distributions of plant species (Violle *et al*., 2012) and how variations in plant strategies can cascade into ecosystem functioning such as decomposition, above‐ and belowground productivity and microbial activities (Suding *et al*., 2008; Lavorel & Grigulis, 2012). However, how plant functional strategies shift in local communities under ongoing climate change, and how strong these shifts are linked to ecosystem functions, is less clear.

Functional traits are not independent of each other. At the individual level, physiological trade‐offs arise from the allocation of limited resources. Accordingly, at the species level, only a few plant functional strategies (i.e. successful trait combinations) along these essential trade‐offs have shown to be evolutionarily viable in vascular plants (Díaz *et al*., 2016). Most studies supporting the idea of a few key trade‐offs in plant functional trait variability focused on large spatial scales, including stark environmental gradients. However, trade‐offs are not robust across environments (Ren *et al*., 2022). Under climate change; they may weaken, affording plants greater flexibility to rapidly adjust to new conditions and potentially enabling the emergence of novel strategies that were absent in previous conditions (Cui *et al*., 2020; Li *et al*., 2022; Wei *et al*., 2023). Thus, if we are to use functional strategy shifts to better understand and early recognize vegetation responses to climate change, we need to establish these trade‐offs at small spatial scales while taking into account plastic responses. Moreover, it is at the community level, in which both individuals' trait changes and species' losses and gains play a role, that we can directly link the functional strategies to ecosystem functions (Mouillot *et al*., 2011; Funk *et al*., 2017; He *et al*., 2023). Whether or not species‐level trade‐offs and functional strategies scale up to the community level may depend on the underlying community assembly processes (Read *et al*., 2014; Rosbakh *et al*., 2015; Chalmandrier *et al*., 2017), and evidence is variable (Funk & Cornwell, 2013; Anderegg *et al*., 2018).

**Fig. 1:**
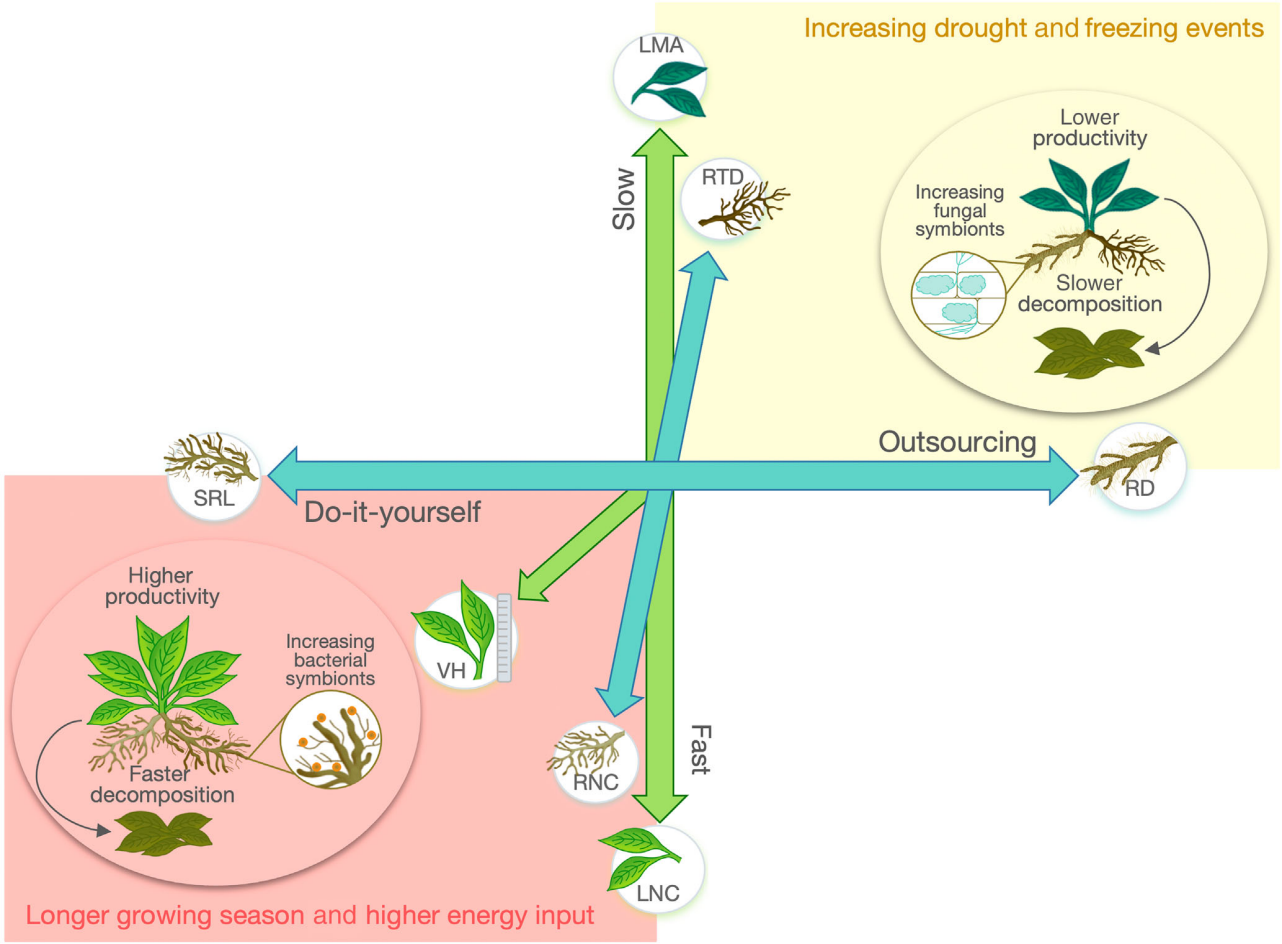
Conceptual framework of plant aboveground (in green) and belowground (in blue) functional trait trade‐offs, shifts in associated plant strategies under climate change and hypothesized links to ecosystem functions and microbial communities. According to the leaf economics spectrum, plants invest either in low LMA and high LNC, the fast‐growing strategy or in opposite traits and a slow‐growing strategy. Plant size constitutes another dimension characterizing plants with higher growth capacity and competitiveness for light. According to the root economics space, along one axis, plants either invest in low RTD and high RNC, the fast resource acquisition strategy or in opposite traits and a slow resource acquisition strategy. Perpendicular to the fast‐slow trade‐off, the trade‐off between RD and SRL characterizes plants' preference to outsource the resource uptake through mycorrhizal associates or a do‐it‐yourself strategy. Note that the debate continues if RTD aligns with the RD‐SRL trade‐off and if leaf and root economics spectra constitute a whole plant economics space. Here, we expect that with climate change, these plant functional strategies shift at the community level through both individuals' trait changes and species' turnover. These shifts will depend on the limiting conditions in the ecosystem. If climate change is providing more benign conditions for plants (red box), for example, through the higher energy input over the year, then we can expect to see a shift toward taller plants with faster and/or more do‐it‐yourself strategies followed by enhanced ecosystem productivity, decomposition rates and a shift toward increasing bacterial activity as both plant symbionts and saprophytes, the latter further enhancing the labile litter decomposition. If climate change is leading to increasing drought and early spring freezing events, then we can expect to see a shift toward shorter plants with slower and/or outsourcing strategies followed by a decrease in productivity and decomposition rates and a shift toward increasing fungal activity as both plant symbionts and saprophytes, the latter further enhancing the recalcitrant litter decomposition. Note that, because the plant economics space and the root collaboration spectrum are orthogonal, all combinations of strategies can theoretically occur across the quadrants (e.g. a fast and outsourcing strategy). For simplicity, we present separate hypotheses related to climate‐driven shifts in functional strategies across all trade‐offs together without violating their orthogonality. References linked to the hypotheses can be found on Supporting Information Table S1. LMA, leaf mass per area; LNC, leaf nitrogen content; RD, root diameter; RNC, root nitrogen content; RTD, root tissue density; SRL, specific root length; VH, vegetative height.

The first trade‐off identified from leaf traits at the species level and at large spatial scales was the leaf economics spectrum (Wright *et al*., 2004; Reich, 2014), as it can be interpreted through economic theory with nutrients, carbon and water as resources, rather than money. Plants invest either in low leaf mass per area (LMA) and high leaf nitrogen content (LNC) – a fast‐growing but short‐lived strategy – or in leaf traits at the opposite end of the spectrum – a slow‐growing but long‐lived strategy. The ‘fast’ strategy improves plant fitness under benign environmental conditions and on fertile soils, while the ‘slow’ strategy is better adapted to stressful conditions such as challenging climates at high altitudes (Fig. 1; Supporting Information Table S1). Plant vegetative height was suggested as a second dimension, with some species investing in early reproduction and staying small and others in becoming tall and thus in strong competitiveness for light (Price *et al*., 2014). Until recently, belowground traits were largely neglected in the description of plant strategies as data are more difficult to acquire. After the root economics spectrum was defined by Freschet *et al*. (2010) and Reich (2014), Bergmann *et al*. (2020) revealed the root economics space through a global root dataset and incorporated root traits into large‐scale analyses of plant functional trade‐offs (Fig. 1). Along the fast‐slow trade‐off, plants exhibit high root nitrogen content (RNC) and low root tissue density (RTD), representing ‘fast’ strategies. Conversely, ‘slow’ strategies are characterized by traits at the opposite end of the spectrum. Additionally, they revealed another root‐specific trade‐off, orthogonal to the root fast‐slow trade‐off, between ‘do‐it‐yourself’ and ‘outsourcing’ strategies for resource uptake (i.e. nutrients and water): At the endpoint of this collaboration spectrum, plants exhibit either high specific root length (SRL) for direct uptake or wider root diameters (RD) for arbuscular mycorrhizal symbiosis. The former is advantageous in resource‐rich soils, while the latter benefits plants under resource scarcity or during drought or freezing events (Augé *et al*., 2015; Laughlin *et al*., 2021). Recent debates question the independence of RTD from the collaboration spectrum (Kong *et al*., 2019; McCormack & Iversen, 2019) and whether a fast vs slow trade‐off in root traits suggests distinct plant strategies (Carmona *et al*., 2021; Bueno *et al*., 2023; Weigelt *et al*., 2023) or aligns with a broader plant economics space (Poorter *et al*., 2014; Weigelt *et al*., 2021), with answers even more elusive at the community level.

Under accelerating climate change in the European Alps, characterized by rising temperatures and more frequent summer droughts (Kotlarski *et al*., 2022; Scherrer *et al*., 2022), we anticipate changes in aboveground and root plant functional strategies at community level – through changes in individuals' traits and community shifts including species' relative abundance changes and turnover – as well as impacts on ecosystem functions and microbial activities (Fig. 1) (Stemkovski *et al*., 2025). However, shifts in plant functional strategies will depend on the plasticity allowed by the strength of the trade‐offs and selection of species demonstrating viable functional strategies under ecosystems' limiting resources. In fact, a recent review on climate change effects showed that shifts in leaf traits depend on whether water is limiting in the system (Myers‐Smith *et al*., 2019). On the one hand, climate warming increases growing season lengths and energy input. Without drought, this may lead to a shift toward taller plants with faster and/or more do‐it‐yourself functional strategies (Fig. 1; references at Table S1). This transition may enhance ecosystem productivity (Bektaş *et al*., 2021) and decomposition rates as, for example, the litter becomes more nutrient‐rich and easily decomposable (Hagedorn *et al*., 2019). Changes in plant communities can cascade to both plant‐associated (e.g. mycorrhizal fungi and nitrogen‐fixing bacteria) and soil microbial communities (e.g. decomposers) through multiple pathways (de Vries *et al*., 2012; Fahey *et al*., 2020; Mészárošová *et al*., 2024). On the other hand, bacterial abundance may increase in communities dominated by fast‐growing plant species, which tend to favor bacterial symbionts such as nitrogen‐fixing and phosphate‐mobilizing bacteria (Henneron *et al*., 2020; Zhou *et al*., 2021). Similarly, in communities adopting do‐it‐yourself nutrient acquisition strategies, bacterial abundance may rise due to reduced competition from fungal symbionts, particularly mycorrhizal fungi (Wang & Kuzyakov, 2024). In parallel, faster growing plants also tend to produce higher quality litter, which can stimulate the activity of saprotrophic bacteria and other decomposer microorganisms. These two distinct pathways – symbiont‐ and litter‐quality‐driven – can together lead to an overall increase in bacterial abundance and a higher representation of copiotrophic organisms (Fierer *et al*., 2007), signaling a shift toward a ‘bacterial energy channel’ (Esperschütz *et al*., 2011) with a fast‐growth strategy of the microbial community. By contrast, increased drought and reduced snow cover during freeze periods may potentially lead to shorter plants with slower and/or more outsourcing strategies (Fig. 1; references at Table S1). Such shifts may impede ecosystem productivity and decomposition rates due to nutrient‐scarce and less decomposable litter. Plant communities characterized by slower growth may invest less in bacterial symbionts, decreasing competition for fungi symbionts (Wang & Kuzyakov, 2024). Moreover, plants with thicker roots promote greater mycorrhizal colonization, further enhancing plant‐associated fungal activity (Ma *et al*., 2018). Increase in recalcitrant litter may enhance a ‘fungal energy channel’ and oligotrophic organisms indicating a shift toward a slower strategy for microbial communities (Pugnaire *et al*., 2019; Osono, 2020). Finally, changes in nutrient availability driven by shifts in microbial energy channels can create feedback loops that reinforce plant functional strategies (Bennett & Klironomos, 2019). Although disentangling the specific underlying processes (e.g. species turnover, litter content or soil community competition) lies beyond the scope of this article, we quantify the indirect effects of climate‐driven shifts in plant functional strategies on the proxies of ecosystem functions and microbial activity.

Here, we aimed at quantifying shifts in above‐ and belowground plant functional traits, ecosystem functions and microbial activities under experimental climate change in mountain grasslands. More specifically, we asked the following: (1) Can we disentangle fast vs slow and do‐it‐yourself vs outsourcing strategies at the community level along an elevational gradient? (2) How do functional traits change under experimental climate change, including warming and drying? (3) How do experimental climate change‐induced changes in functional traits link to the above‐ and belowground ecosystem functions? We answered these questions by simulating *in situ* climate change on alpine intact plant and soil communities by transplanting them to lower elevations.

## Materials and Methods

### Study site and experimental design

To simulate climate change, that is climatic warming and drought, in the French Alps, we moved alpine grassland plots from high elevation to *c*. 500 m lower elevation in September 2016 (Bektaş *et al*., 2021). The alpine site ‘Galibier’ (45°05′44″N, 06°40′06″E at 2450 m altitude) and the subalpine site ‘Lautaret’ (45°04′00″N, 06°41′90″E at 1920 m altitude) were close to each other (*c*. 2 km airline distance), had a similar orientation (South‐East at alpine and South–South‐West at subalpine site), bedrock (base‐riched flysch) and soil (dystric cambisols). This system represents a relatively dry mountain grassland, and both alpine and subalpine zones are generally subject to water limitation (Leitinger *et al*., 2015). At the alpine site, communities were dominated by *Trifolium alpinum* L., *Potentilla aurea* L., *Plantago alpina* L., *Carex sempervirens Vill. subsp. sempervirens* and *Poa alpina* L. At the subalpine site, communities were dominated by *Patzkea paniculata* (L.) *G. H. Loos subsp. paniculata*, *Festuca nigrescens Lam*., *Carex sempervirens Vill. subsp. sempervirens*, *Festuca laevigata Gaudin*, and *Plantago maritima subsp. serpentina* (*All*.) *Arcang*. (the complete list of species is given in Table S2). We transplanted 10 replicates of 4 m^2^ plots of vegetation with at least 20 cm of their intact belowground parts and the surrounding soils from the alpine to the subalpine site (AlpineWarmed plots). For the transport, each turf was cut in 4 × 1 m^2^ pieces and then reassembled in their original composition when replanted. Around the turfs, we placed water‐permeable root barriers to avoid root ingrowth from the natural vegetation. To control for the transplantation stress, we also horizontally transplanted 10 intact 1 m^2^ blocks at the alpine site (AlpineControls) and at the subalpine site (SubalpineControls), respectively. At both sites, cattle and sheep were excluded by electric fences, and biomass was not removed after each growing season. More details on the experimental design can be found in Bektaş *et al*. (2021).

**Fig. 2:**
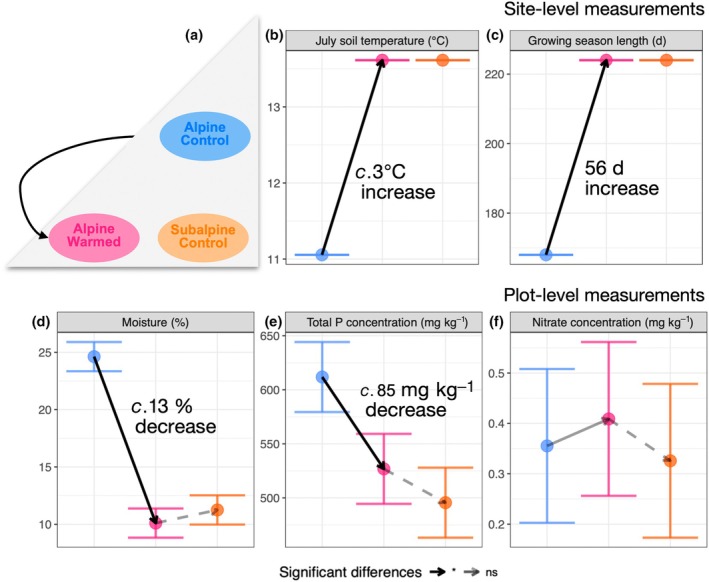
Change in climatic conditions and soil nutrients 5 yr after transplantation of alpine communities from high elevation to low elevation (a). Under experimental climate change, an increase in temperature and growing season length might create more benign conditions for plants (b, c). However, as the snow melts earlier, plants may be exposed to early and more frequent spring freezing events. A decrease in soil moisture (d) and in soil phosphorus (e) may create water and nutrient scarcity for plants, while (f) nitrogen shows no significant change and a tendency to increase in transplanted plots. The direction of the arrows indicates an increase or a decrease with the transplantation of alpine communities to subalpine sites. The acclimation lag after experimental climate change is shown in black dashed lines. Nonsignificant differences are shown in transparent lines. ns, nonsignificant. Error bars represent 95% confidence intervals.

In 2021, the year of data collection, average July soil temperature was 3°C warmer at the subalpine site than at the alpine site, and the snow‐free season was 56 d longer, corresponding to a 33% increase (Fig. 2b,c). In each plot, we measured the soil moisture 21 times during the growing season (from mid‐June to August) with Campbell Scientific HydroSense II and took the median soil moisture per plot. The average summer soil moisture was significantly lower in AlpineWarmed than in AlpineControl plots (difference between AlpineWarmed and AlpineControl: 12.73 ± 1.05%; Fig. 2d). In 2020, we also measured soil nutrients of each plot. A 0.5 M K_2_SO_4_ solution was used to extract soil nitrate (NO_3_^−^), ammonium (NH_4_^+^), total dissolved nitrogen (Jones & Willett, 2006) from 10 g of fresh soil. Nitrogen (N) concentrations were determined using an automated photometric analyzer (Gallery Plus, Thermo Fisher Scientific, Waltham, MA, USA) following standard colorimetric methods. Total phosphorus was extracted from 0.15 g of dry soil when available, using a modified EPA 3050B protocol involving successive acid digestion: Samples were heated at 95°C with 30 ml of concentrated HNO_3_ for 3 h, followed by 2 ml of H_2_SO_4_ for 2 additional hours. Phosphorus concentrations were determined colorimetrically using the molybdenum blue method. Soil nitrate concentration as the plant‐available nitrogen did not differ between AlpineControl and AlpineWarmed plots (Fig. 2f), but total soil P concentration decreased significantly with experimental climate change (difference between AlpineWarmed and AlpineControl: 84.97 ± 23.43 mg P kg^−1^; Fig. 2e).

### Community‐level leaf traits

We measured community‐level leaf traits *in situ* by sampling individuals with a 5‐cm distance along two diagonals of a 1 m^2^ part of each plot and thus 29 individuals in each plot (290 individuals sampled per experimental treatment). Since sampling occurred at the intersection of a 29‐point ruler and a metal pin, a single plant usually made contact. In rare cases of multiple leaves touching, we selected the one with the greatest surface contact. This method naturally takes into account the coverage of the species as there is a higher probability of sampling species with higher coverage. We measured the vegetative height (cm), LMA (kg m^−2^) and LNC (without petiole) (mg g^−1^) based on the standardized protocol from Cornelissen *et al*. (2003). Of the 82 species sampled across all experimental groups, 79% are arbuscular mycorrhizal, while 15% can be either mycorrhizal or nonmycorrhizal. The species composition includes 67% forbs, 27% graminoids, 5% legumes and 1% shrubs. Among legumes, *Trifolium alpinum* L. is abundant in AlpineControl and AlpineWarmed communities, while *Trifolium montanum* L., *Trifolium pratense* L., and *Trifolium alpestre* L. occur in SubalpineControl. Due to limited sampling material, LNC measurements were not possible for 0.3% of individuals in AlpineControl, 2.1% in AlpineWarmed and 1% in SubalpineControl. Additionally, vegetative height data for one species was missing in both AlpineWarmed and SubalpineControl plots. None of the missing data were biased toward a specific species. After evaluating data quality, we excluded one LNC measurement from AlpineWarmed communities (Notes S1; Figs S1, S2). We calculated community‐level leaf traits as the mean of log‐transformed, standardized leaf traits for all sampled individuals per plot.

### Community‐level root traits

We measured the community‐level root traits *in situ* by collecting the fine roots with root ingrowth cores (Freschet *et al*., 2021). In September 2020, we planted two soil ingrowth cores (15 cm deep, 5 cm diameter, plastic mesh size of 3 mm) in each plot. The ingrowth cores were filled with river sand as a standardized root ingrowth medium, let 1 yr in place and sampled in September 2021 by merging the two cores of one plot into a single ziplock bag. Directly after sampling, we (1) washed the samples to separate fine roots from sand, (2) weighed the fresh mass of the fine roots, (3) scanned them to obtain measures of RD, length and volume (WinRhizo software), (4) dried them for 72 h at 65°C and weighed the dry mass and (5) measured RNC (mg g^−1^) on dry and ground subsamples using an elemental analyser (FlashEA 1112; Fisher 302 Scientific, Waltham, MA, USA). We calculated SRL as the ratio of root length to root dry mass (m g^−1^) and RTD (g cm^−3^) as the ratio of root dry mass to volume (Freschet *et al*., 2021). We log‐transformed and standardized the root traits. Root cores in one SubalpineControl were missing. Hence, we gap‐filled the values with the mean of the nine other replicates of SubalpineControl plots.

### Ecosystem functions and soil microbial activities

We focused on above‐ and belowground ecosystem functions that are tightly linked to plant communities: above‐ and belowground plant productivity, decomposition of standardized recalcitrant (rooibos tea) and labile (green tea) litter (Keuskamp *et al*., 2013). We used dry mass of fine roots as a proxy of annual belowground biomass. As a proxy of annual aboveground biomass production, we measured in each plot in each week of the growing season the Normalized Difference Vegetation Index (NDVI) with Decagon ProCheck spectral reflectance sensor, and estimated the area under the NDVI curve from the snowmelt to snowfall in 2021 (Bektaş *et al*., 2021). Green (labile) and red (recalcitrant) teabags (Keuskamp *et al*., 2013) were installed for 1 yr beneath the soil surface in each plot close to the soil ingrowth cores in September 2020, sampled in September 2021 and dried for 48 h at 70°C to measure their dry mass. From this, we calculated the decomposition rate as the difference of the initial dry mass of the teabag and the measured dry mass in 2021 divided by the initial dry mass. We used the decomposition rates of labile and recalcitrant standard litters as proxies for both the overall pace of decomposition and the decomposition activity of bacterial energy channels or copiotrophic soil organisms and fungal energy channels or oligotrophic soil organisms, respectively.

We also identified the root‐associated microbial communities via measuring the percentage of arbuscular mycorrhizal colonization in the roots and the number of 16S rRNA gene copies per root mass (g) as a proxy for bacterial biomass. Higher bacterial biomass in a given experimental group than others indicated stronger bacterial activity and thus faster microbial activities of fast‐growing microbial communities. Higher mycorrhizal colonization in a given experimental group than others indicated stronger collaborative microbial activities. We quantified arbuscular structures within cleaned and stained root samples (Vierheilig *et al*., 1998, 2005). To ensure equal sampling, we prepared slides from 1‐cm segments of 10 roots per community sample. Each segment was examined at three randomly chosen intersections, totaling 30 observation points per sample. To mitigate observer bias, these observations were independently replicated by three observers. Arbuscular colonization was then quantified as the median percentage of observed arbuscular presence across the three observers. Finally, we performed quantitative PCR (qPCR) on dry root samples (i.e. related protocol specified at Notes S2). We log‐transformed and standardized all measurements.

### Statistical analyses

To disentangle functional plant strategies, we built a principal component analysis (PCA) on leaf (LMA, LDMC, LNC) and root (i.e. SRL, RD, RNC, RTD) traits related to our two focal trade‐offs measured in control plots (Fig. 1). We conducted a varimax rotation on the first two axes of this PCA to be able to assure better interpretability of the components and of the trait loadings on the first two components (Carmona *et al*., 2021).

Communities acclimate to new climate conditions after transplantation through two processes: changes in individuals' traits as well as community shifts including species' relative abundance changes and turnover. However, note that the species turnover, especially through the establishment of the surrounding subalpine species in the AlpineWarmed communities, is low (Notes S3; Figs S3, S4). Thus, to investigate the effect of experimental climate change on the plant functional traits at the community level, ecosystem functions and soil microbial activities, we calculated the experimental climate change effect (difference between AlpineWarmed and AlpineControl), community‐level acclimation lag after experimental climate change (difference between SubalpineControl and AlpineWarmed) for each variable using ANOVAs (Tables S3–S5) (Bektaş *et al*., 2021). While the experimental climate change effect quantified the amount of change after transplantation, acclimation lags indicated how much change was further needed to reach full acclimation under the assumption that the control communities are at a stable state and fully acclimated to their current conditions (a more elaborated discussion can be found at Bektaş *et al*., 2021). We adjusted the *P*‐values with multivariate *t*‐distribution adjustment for multiple tests.

We additionally investigated which change in the environmental conditions the transplanted alpine communities respond to. For this, we conducted a redundancy analysis (RDA) with the functional traits as response variables and with site‐level soil temperature (i.e. single value for all plots in each site reflecting the treatment effect), plot‐level soil moisture and plot‐level soil nutrients (total phosphorus and nitrate concentration) as explanatory variables. RDA performs multiple linear regressions of multiple response variables followed by a PCA of the fitted values (Legendre & Legendre, 2012). RDA allows us to test the overall impact of different environmental conditions on all of the functional traits simultaneously. We tested the significance of the RDA model, RDA axes and explanatory variables with permutation tests (i.e. 1000 permutations of the plots; Table S6) (Borcard *et al*., 2011). We concluded that the significant explanatory variable with the most proportion of the explained variance has the strongest impact on the functional traits.

Finally, to assess the changes in the links between the community‐level functional traits and ecosystem functions and microbial activities with experimental climate change, we conducted a RDA with the ecosystem functions and microbial activities as response variables, and with functional traits, experimental climate change (i.e. 0/1 for controls and AlpineWarmed, respectively) and their two‐way interaction as explanatory variables. Here, we included both controls because we hypothesized that even if the functional traits and ecosystem functions change from high to low elevation under control conditions, the link (i.e. the correlation) between them should remain the same, but it should change (i.e. temporarily broken or sign of the correlation changes) under experimental climate change. We tested this with an RDA, and we chose to perform RDA because we did not want to assume a causal one‐way relationship between functional traits, ecosystem functions and microbial activities, as there are feedbacks within and between them. Because of the high correlation between labile and recalcitrant litter decomposition rates (*r* = 0.49, *P* < 0.01), we only included the labile litter decomposition rate in the analysis. We tested the significance of the RDA model, RDA axes and explanatory variables with permutation tests (i.e. 1000 permutations of the plots; Table S7). When a given functional trait was only significant without its interaction with the experimental climate change, we concluded that the functional trait has a strong link to ecosystem functions, but the links do not change from controls to experimentally changed conditions. When only the experimental climate change effect was significant, we concluded that there were experimental climate change effects that we could not explain with the functional traits. When the interaction between a given functional trait and experimental climate change was significant, we concluded that the links (i.e. magnitude or direction) between functional traits and ecosystem functions and microbial activities change with the experimental climate change. When the variation explained by the interaction between a functional trait and experimental climate change was as high as that explained by the trait alone, we interpreted this as a tendency for the relationship (i.e. magnitude or direction) between the trait and ecosystem functions or microbial activities to shift under experimental climate change.

We conducted the statistical analyses with R v.4.4.1 (R Core Team, 2024) with the following packages: emmeans (Lenth, 2022), vegan (Oksanen *et al*., 2022), psych (William Revelle, 2024).

## Results

### Can we disentangle functional trait trade‐offs in control plots?

**Fig. 3:**
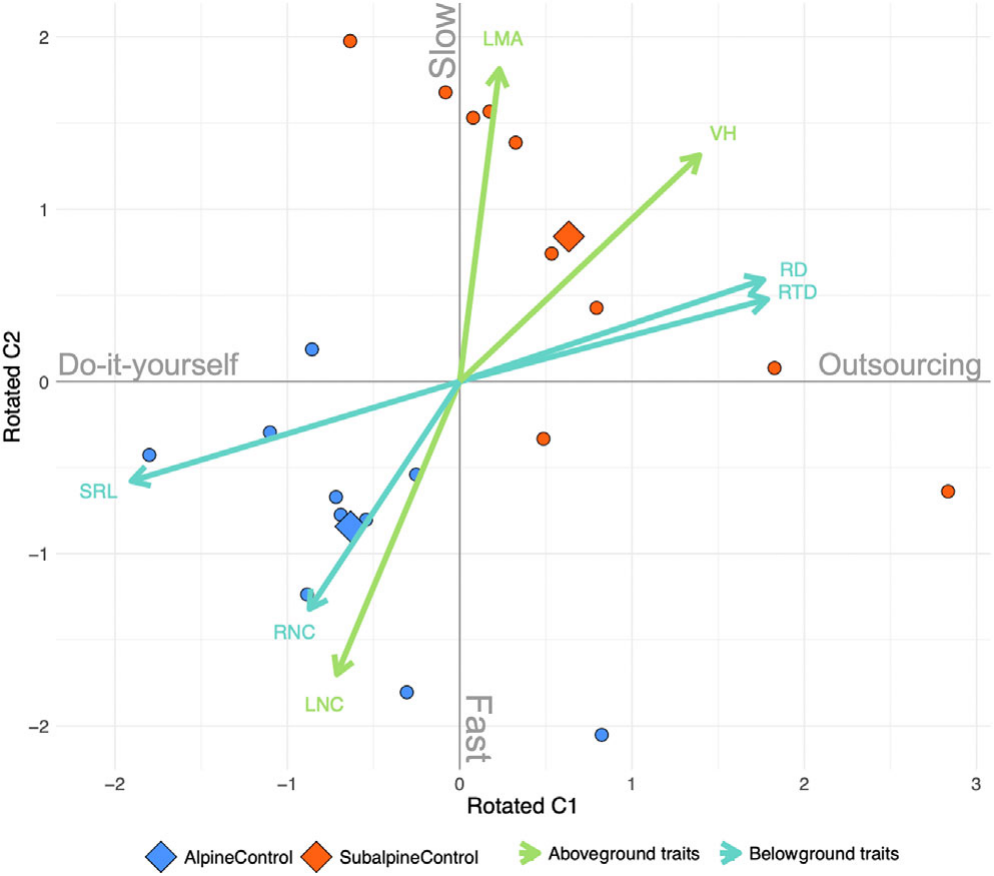
Community‐level functional trait space constructed with a principal component analysis followed by a varimax rotation with fast vs slow and do‐it‐yourself vs outsourcing trade‐offs of the control plots (circles). Fast vs slow trade‐off is constructed from LNC, RNC and LMA. Do‐it‐yourself vs outsourcing is constructed from SRL, RD and RTD. Diamond shapes indicate the centroids of the control plots. LMA, leaf mass per area; LNC, leaf nitrogen content; ns, non‐significant; RD, root diameter; RNC, root nitrogen content; RTD, root tissue density; SR, specific root length; VH, vegetative height.

**Fig. 4:**
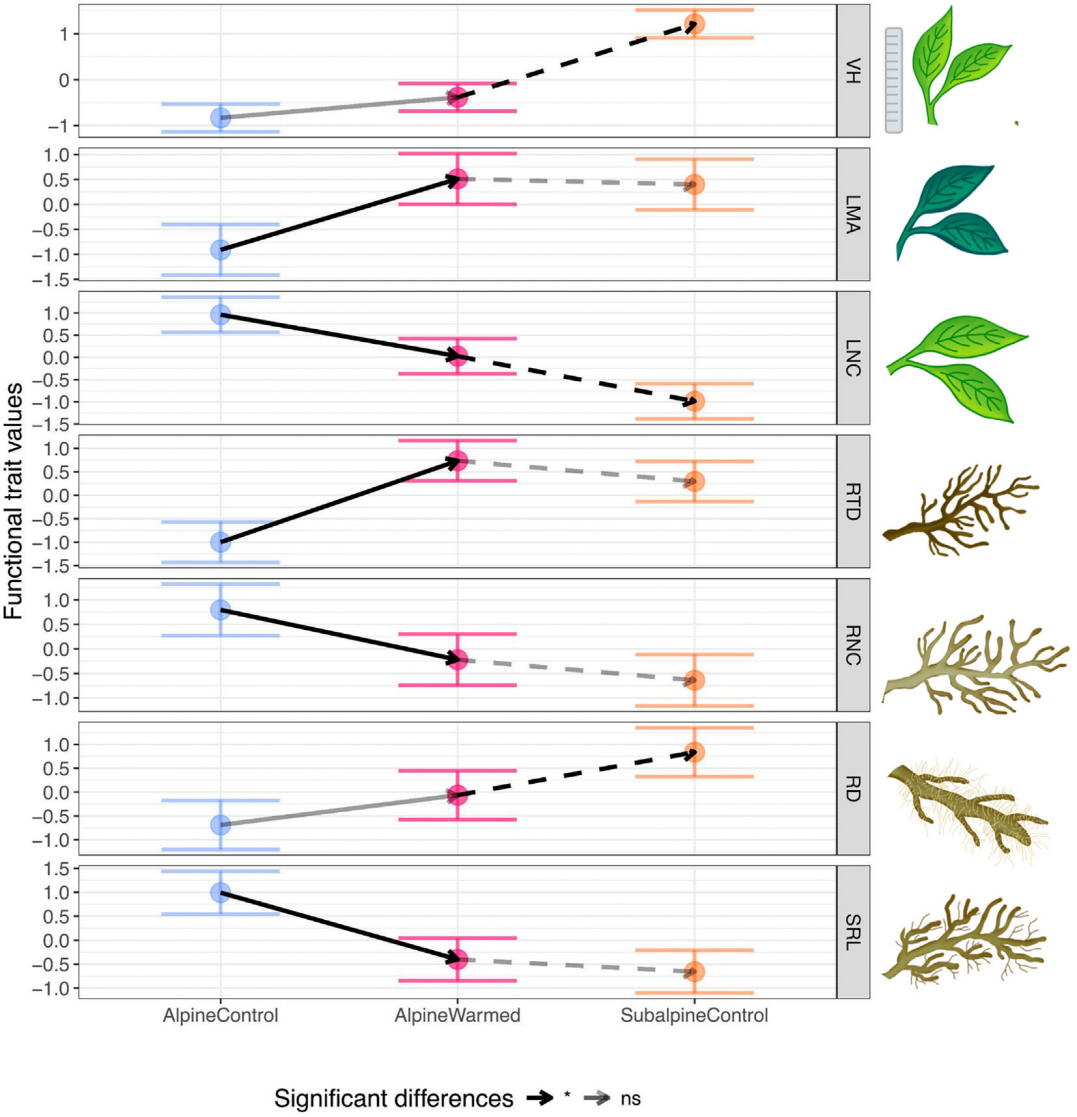
Community‐level functional trait values of experimental plots. The experimental climate change effect (in black solid lines) is the difference between AlpineWarmed and AlpineControl plots. The direction of the arrows indicates an increase or a decrease with the experimental climate change effect. The acclimation lag after experimental climate change (in black dashed lines) is the difference between the AlpineWarmed and SubalpineControl plots. Nonsignificant differences between experimental plots are transparent. Error bars represent 95% confidence intervals. Trait values are log‐transformed and z‐score normalized. A visualization of nontransformed values can be found in Supporting Information Fig. S5. LMA, leaf mass per area; LNC, leaf nitrogen content; ns, nonsignificant; RD, root diameter; RNC, root nitrogen content; RTD, root tissue density; SRL, specific root length; VH, vegetative height.

The first two axes of the PCA linked to the fast vs slow and the do‐it‐yourself vs outsourcing trade‐offs (Fig. 3) explained 84% of the variation in functional trait space. After the varimax rotation, SRL and RD mainly loaded on the first rotated axis (loadings: −0.95, 0.88, respectively) representing the do‐it‐yourself vs outsourcing trade‐off, while LMA, LNC and RNC mainly loaded on the second axis (loadings on: 0.92, − 0.81, −0.64, respectively) representing the fast vs slow trade‐off (Table S8). Thus, we showed that the hypothesized trade‐offs (Fig. 1) partially occurred at the community level in sites on the same mountain slope and thus along a short environmental gradient. There were two exceptions: RTD mainly loaded on the first axis (loading: 0.89) together with SRL and RD (Fig. 3; Table S8), and vegetative height did not constitute a third orthogonal axis of plant size but equally loaded on the two axes (Table S8). AlpineControl communities exhibited a faster and more of a do‐it‐yourself strategy than SubalpineControl communities (Figs 3, 4).

### How do functional traits change under experimental climate change?

**Fig. 5:**
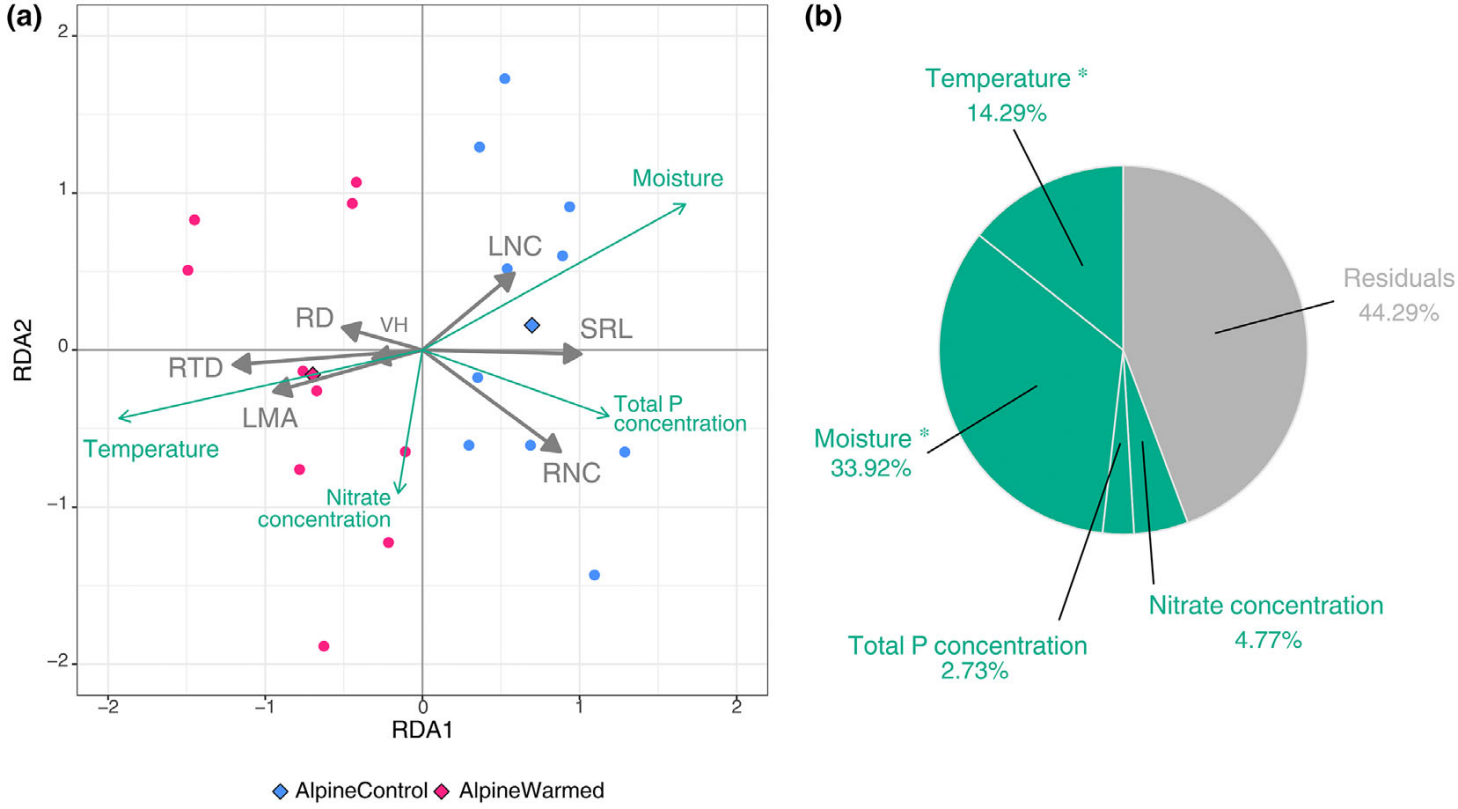
Linking community‐level functional traits to environmental conditions. (a) Redundancy analysis (RDA) between functional traits and environmental conditions as induced by the experimental climate change. Gray and colored arrows indicate response and explanatory variables, respectively. Centroids of the experimental plots are indicated in diamond shaped points. The RDA correlation triplot is constructed with scaling = 2 meaning that the angles between variables reflect correlations between them. (b) Proportion of variance explained by each variable. Significant variables are indicated with a star (*). Note that the soil temperature was measured at site level (i.e. single value for all plots in each site), whereas all the other variables at plot level. LMA, leaf mass per area; LNC, leaf nitrogen content; ns, nonsignificant; RD, root diameter; RNC, root nitrogen content; RTD, root tissue density; SRL, specific root length; VH, vegetative height.

Just like SubalpineControl communities, AlpineWarmed communities exhibited slower and more outsourcing strategies than AlpineControl communities (Fig. 4). Experimental climate change effects on traits related to the fast vs slow trade‐off included an increase in LMA and a decrease in LNC and RNC (Fig. 4). Experimental climate change effects on traits related to the do‐it‐yourself vs outsourcing trade‐off included an increase in RTD and a decrease in SRL (while the increase in RD was not significant). Moreover, vegetative height tended to increase under experimental climate change, but the effect was not significant. Interestingly, for LMA, RTD, RNC and SRL, we did not observe significant acclimation lags of communities; that is, differences between SubalpineControl and AlpineWarmed plots, with the exception of VH (smaller in AlpineWarmed than in SubalpineControl; Fig. 4), LNC (higher in AlpineWarmed than in SubalpineControl) and RD (narrower in AlpineWarmed than in SubalpineControl). The RDA shows that functional traits responded the most not only to the changes in soil moisture (34% of explained variance in functional traits) but also to the changes in temperature (14% of explained variance in functional traits) under experimental climate change (Fig. 5).

### Cascading effects from experimental climate change via functional traits to ecosystem functions and microbial activities?

**Fig. 6:**
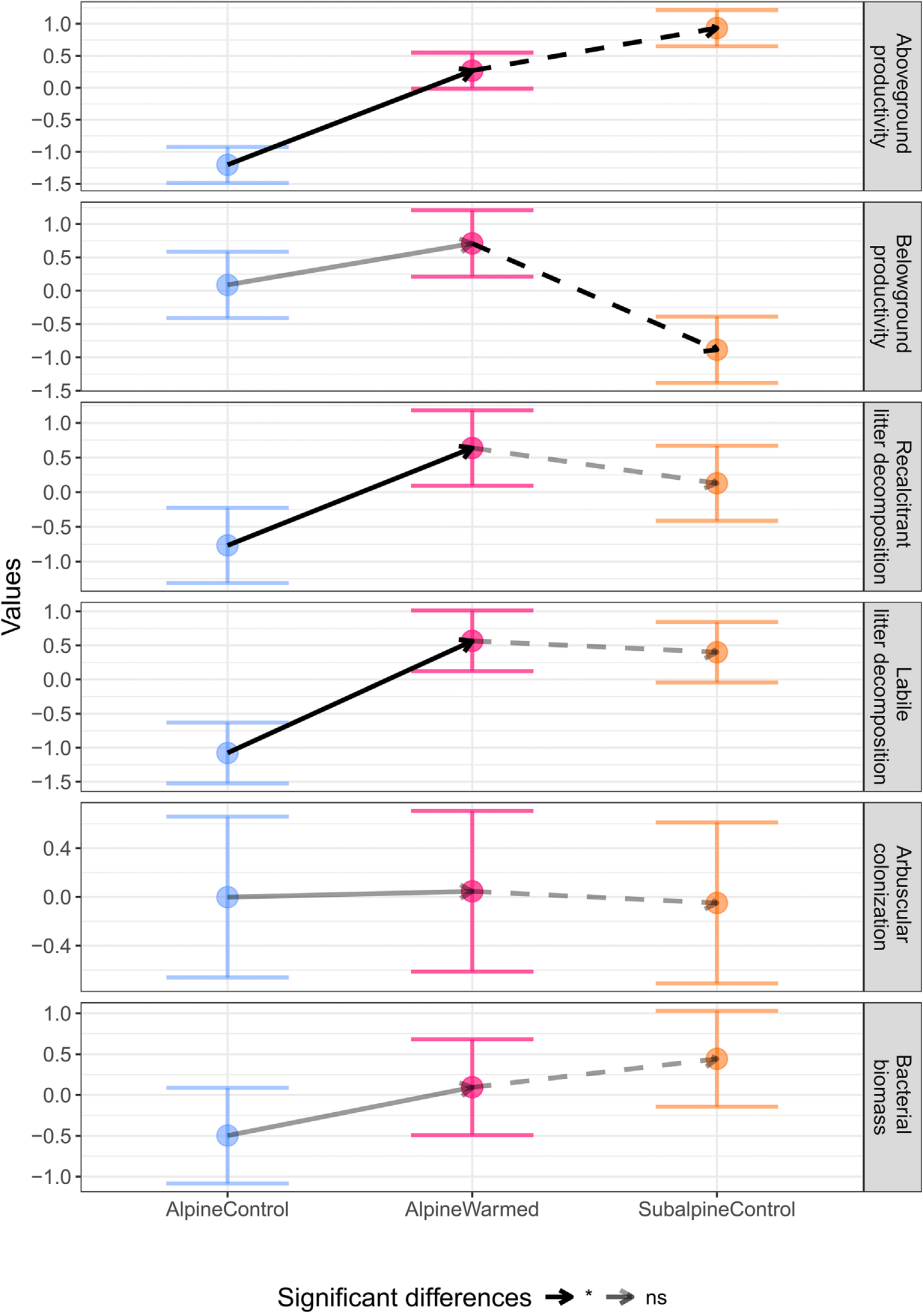
Ecosystem functions and microbial activities of the experimental plots. The experimental climate change effect (in black solid lines) is the difference between AlpineWarmed and AlpineControl plots. The direction of the arrows indicate an increase or a decrease with the experimental climate change. The acclimation lag after experimental climate change (in black dashed lines) is the difference between the AlpineWarmed and SubalpineControl plots. Nonsignificant differences between experimental plots are transparent. Values are log‐transformed and *z*‐score normalized. Error bars represent 95% confidence intervals. A visualization of nontransformed values can be found in Supporting Information Fig. S6. ns, nonsignificant.

Under experimental climate change, all ecosystem functions and microbial activities were enhanced, with significant increases in aboveground productivity and decomposition rates and nonsignificant increases in belowground productivity, arbuscular colonization and bacterial biomass (Fig. 6). Decomposition rates fully acclimated, while the increase in aboveground productivity was not enough to reach full acclimation (i.e. AlpineWarmed productivity was significantly lower than SubalpineControl). Interestingly, belowground productivity also tended to increase (i.e. even though nonsignificantly) with experimental climate change, even though under subalpine conditions, SubalpineControl plots have lower productivity than AlpineControl plots.

**Fig. 7:**
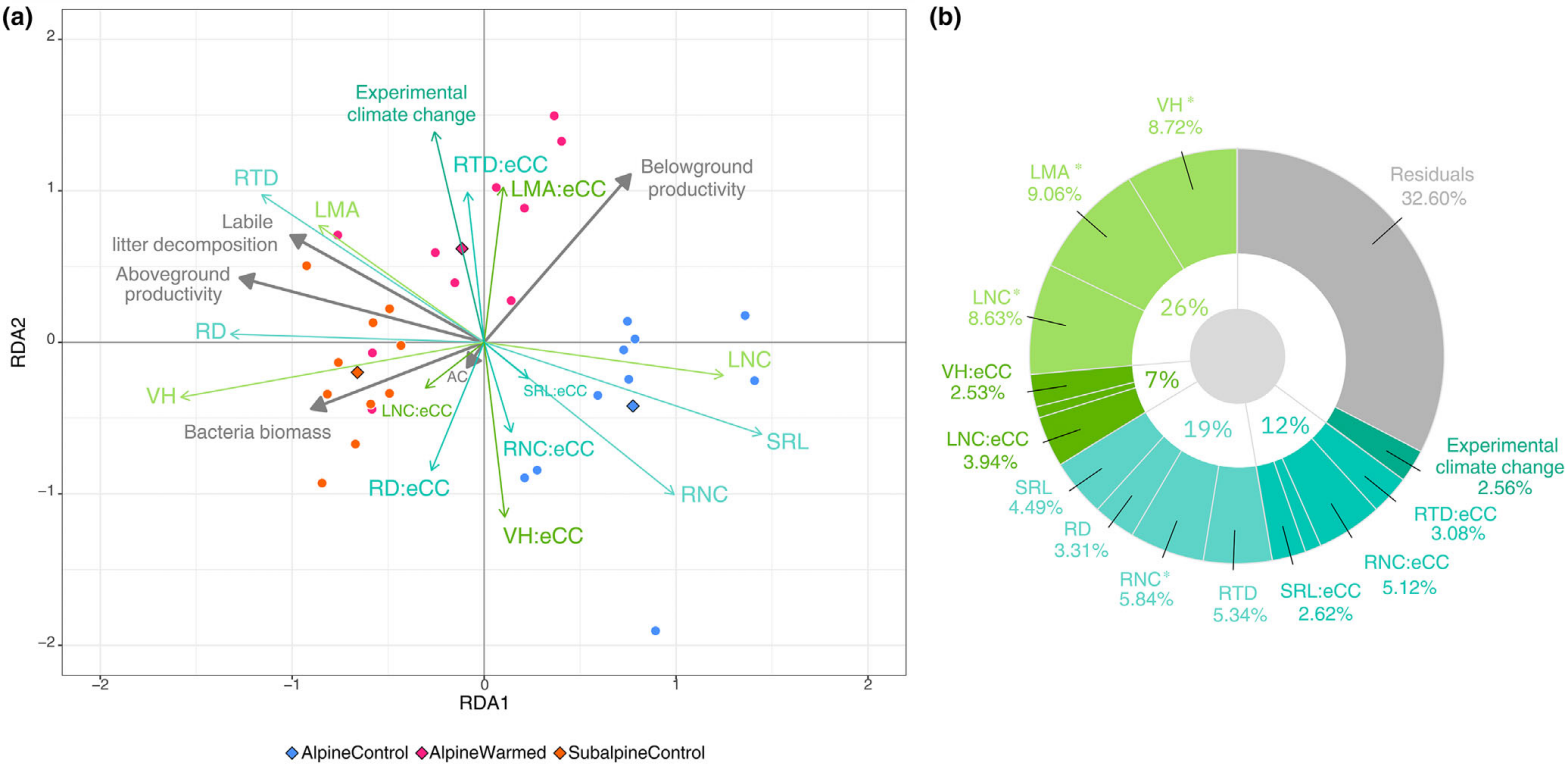
Linking ecosystem functions to functional traits, experimental climate change and their interaction. (a) Redundancy analysis between ecosystem functions (response variables: litter decomposition, above‐ and belowground productivity, bacterial biomass) and explanatory variables, including functional traits, experimental climate change and their two‐way interactions (denoted ‘:eCC’). Gray and colored arrows indicate response and explanatory variables, respectively in (a). Centroids of the experimental plots are indicated in diamond shaped points. The RDA correlation triplot is constructed with scaling = 2 meaning that the angles between variables reflect correlations between them. (b) Proportion of variance in ecosystem functions explained by each variable. Significant variables are indicated with a star (*). Relative contributions of response and explanatory variables on both axes can be found at Supporting Information Tables S9 and S10. AC, arbuscule colonization; eCC, experimental climate change; LMA, leaf mass per area; LNC, leaf nitrogen content; ns, nonsignificant.RD, root diameter; RNC, root nitrogen content; RTD, root tissue density; SRL, specific root length; VH, vegetative height.

RDA revealed that a large portion of the variation in ecosystem functions and microbial activities (*c*. 70%) was explained by functional traits and experimental climate change effects (Fig. 7). When considered together with their interaction with experimental climate change, above‐ and belowground traits (along with their interactions with experimental climate change) explained comparable amounts of variation in ecosystem functions and microbial activities (Fig. 7b). However, when considered alone (i.e. without their interaction with experimental climate change), aboveground traits had significant effects and accounted for the largest proportion of explained variation. Whereas of the belowground traits, only RNC had a significant effect (Fig. 7; Table S5). While none of the interactions between individual traits and experimental climate change were statistically significant, the interaction of belowground traits with experimental climate change still explained nearly as much variation as these traits alone. This shows that there is a tendency for the links between the belowground functional traits and ecosystem functions and microbial activities to become disrupted. Overall, the experimental climate change alone was not significant and only explained < 3% of the variation in ecosystem functions and microbial activities.

## Discussion

The response of plant communities to climate change is recognized as one of the fundamental mediators of environmental change impacts on ecosystem functions and microbial activities. Here, we studied the responses of plant functional strategies to 5 yr of *in situ* climate change and the cascading effects on related ecosystem functions. We observed strong experimental climate change effects on the functional traits related to two dominant trade‐offs. Nevertheless, communities did not reach full acclimation for either of the trade‐offs. Moreover, we observed that changes in functional traits cascaded to changes in ecosystem functions. Most of the variance in ecosystem functions and in microbial communities was explained by the aboveground functional traits, and links between traits and ecosystem functions did not change under experimental climate change. By contrast, the links between belowground functional traits and ecosystem functions, along with microbial communities, showed a tendency to become disrupted under climate change. Interruptions of such links between plant strategies and ecosystem functions might challenge our capacity to predict the trajectories of plant and soil communities and their associated ecosystem functions under climate change.

### Species' orthogonal trade‐offs upscale to communities

We showed that in a small‐scale community‐level experiment with a relatively short environmental gradient, fast vs slow and do‐it‐yourself vs outsourcing trade‐offs can well explain trait variation. As shown earlier in large‐scale observations on species distributions along stark environmental gradients (Bergmann *et al*., 2020; Weigelt *et al*., 2021), these two dimensions were orthogonal to each other and sufficient to represent plant functional strategies with the exception of root tissue density that aligned rather with the do‐it‐yourself vs outsourcing trade‐off. To our knowledge, there is so far a single study that investigated the presence of orthogonal trade‐offs simultaneously in leaf and root traits at community level in a natural context in which root tissue density did not align with the do‐it‐yourself vs outsourcing trade‐off (Lachaise *et al*., 2022). Here, in addition, we showed that this framework of orthogonal trade‐offs can be particularly useful in determining abiotic drivers of acclimation for plants and studying ongoing climate change on shifts in plant functional strategies.

### Soil moisture determines the functional strategies and their acclimation to climate change in mountain grasslands

One of the questions under climate change (and in our experiment) is whether the increasing temperatures and the associated increased energy input lead to more benign conditions for higher altitude mountain plants, or whether the increased drought, especially during summers, will limit plant growth. In our experiment, we found that aboveground plant productivity in the warmer and drier subalpine sites was higher than in the colder and less dry alpine sites, and productivity of alpine communities increased when they were transplanted to warmer and drier conditions. This indicates that plant productivity overall benefited more from the additional energy input than it suffered from added drought. However, plant functional strategies responded differently. The colder and less dry alpine communities were faster and more do‐it‐yourself than the warmer and drier subalpine communities, and the transplanted alpine communities became slower and more outsourcing after transplantation. These differences were both driven by the differences in species composition (Figs S3, S4) and trait plasticity. Overall, plant functional strategies were more influenced by the additional drought than by the added energy. Such drought responses have been reported earlier (Laughlin *et al*., 2021; Lachaise *et al*., 2022), as mycorrhizas supported plant growth under drought (Augé *et al*., 2015), and communities adopted slow‐growth strategies to withstand drought. Our community‐level results highlighted the sensitivity of the alpine vegetation to drought events that are increasingly frequent under climate change and in line with landscape‐level findings (Choler, 2023). Thus, community‐level responses of functional traits both through plasticity and species turnover to drought even at a local scale can serve as early warning signals of changes in functional composition of alpine plant biodiversity.

### Asynchronous acclimation in functional strategies: transient biotic interactions?

Root traits (except RD) and LMA, important traits in determining plants' resilience to drought (Poorter *et al*., 2009; Blumenthal *et al*., 2020; Laughlin *et al*., 2021), acclimated more efficiently to experimental climate change (i.e. complete resemblance to subalpine communities) than vegetative height, important in determining plants' growth capacity and competitiveness for light (Craine & Dybzinski, 2013). This is perhaps showing that under multiple environmental changes, there is a priority in community‐level plastic responses: Alpine communities primarily dedicate resources to construct more resilient roots and leaves (or such species are selected in the communities) under drought than growing taller and acquiring competitive advantage when there is more energy in the system. This might be a disadvantage for alpine species, especially when these communities are being invaded by taller, thus more competitive (i.e. for acquiring light) subalpine species as they migrate upslope to follow their abiotic niche under future climate change (Lenoir *et al*., 2008). In our experiment, communities were exposed not only to climate change but also to the establishment of new species from the surrounding subalpine communities (Notes S3). Yet, colonization events were not as frequent in our experiment in contrast to other transplant experiments (Bektaş *et al*., 2024). For example, one of the tallest and very abundant species in the subalpine communities, *Patzkea paniculata subsp. paniculata*, did not colonize the warmed alpine communities at all until now (Fig. S4). Colonization lags may occur when seeds fail to establish under a closed canopy. However, we observed several open soil patches in our communities and instances in which colonization occurred from the borders by clonal species. In fact, Bektaş *et al*. (2024) showed with > 40 different whole‐community transplant experiments world‐wide with varying plot sizes and experimental designs that colonization lags persisted at the end of the experiments when the transplanted high‐elevation communities exhibited fast‐growing strategies. In our experiment and beyond, such lags in colonization events might have been due to persistence of fast strategies in alpine communities giving them a competitive advantage in acquiring soil nutrients and water or by maintaining the soil conditions and microbial activity in their favor and preventing the establishment of new subalpine species. In fact, transplanted communities in the Austrian and Swiss Alps, initially exhibiting slow‐growth strategies, were colonized significantly by subalpine species, which caused a complete switch in fast‐growing functional strategies (Schuchardt *et al*., 2023). Together with our results, this shows that the initial functional state of the mountain grassland communities is an important determinant of their functional and taxonomic trajectory under climate change.

### Disruption of belowground functional traits and functions under climate change

In our experimental communities, functional traits explained a large part of the variation of ecosystem functions and microbial activities, suggesting that climate change induced changes in functional traits cascade to ecosystem functions and microbial activities. However, some of the links between functional traits and ecosystem functions were not in line with our expectations. Ecosystem productivity and fast decomposition rates, often associated with fast‐growing plant strategies (Poorter *et al*., 2009; Pietsch *et al*., 2014) and hypothesized to be associated with do‐it‐yourself plant strategies, were associated with slow and outsourcing strategies in our experiment. Interestingly, these relationships were not altered with climate change treatments either. In fact, slow‐growing subalpine communities and warmed alpine communities, despite being exposed to drought, had longer growing seasons than the alpine communities under alpine conditions, and thus, they had the opportunity to grow longer, increasing the community productivity (Bektaş *et al*., 2021). But in comparison under the same warm and dry subalpine conditions, the slower and more outsourcing subalpine communities were more productive than the faster and more do‐it‐yourself alpine communities. Therefore, it seems like there is no clear trade‐off between higher productivity and slow growth in mountain grasslands.

Under experimental climate change, aboveground traits responded strongly but insufficiently (i.e. acclimation lags remained) and links between traits and functions remained unchanged, and aboveground productivity increased, even so, with acclimation lags remaining. By contrast, belowground traits responded strongly and efficiently (except RD). However, the links between traits and ecosystem functions showed a tendency to be altered with climate change, and belowground productivity tended to change in the ‘counterintuitive’ direction (i.e. in a different direction than expected given the subalpine control communities). This may indicate a breaking coordination of below‐ and aboveground plant acclimation potentially due to opposing responses to simultaneously increasing energy input and drought, and with consequences for some ecosystem functions. While aboveground functional traits remain good predictors of ecosystem functions under climate change, this could only be a transient state, and disruptions between belowground traits and ecosystem functions can be early warning signals of acclimation problems.

### Limitations and perspectives

While we demonstrated fast but insufficient acclimation of alpine plant community traits to climate change, a clearer understanding of acclimation lags requires decomposing trait acclimation into plasticity and species turnover. Fully achieving this would require sampling all individuals, which is impractical for long‐term experiments. However, hyperspectral and high‐resolution 3D imagery could estimate aboveground traits efficiently.

Moreover, this study provides only proxies for changes in decomposition rates and microbial activities. Targeted sequencing of arbuscular mycorrhizas, litter‐decomposing and symbiotic bacteria, or full genome sequencing, could offer a more complete picture of plant–soil feedbacks under climate change. Future research should also examine how microbial acclimation lags influence plant community responses. Finally, a global synthesis of 40+ transplant experiments revealed a common trend of rapid convergence toward low‐elevation communities under warming; yet colonization and extinction lags persist (Bektaş *et al*., 2024). Variation in responses was linked to initial climate conditions and functional diversity, underscoring the context dependency of ecological processes. Given the logistical challenges of local experiments, cross‐study analyses within transplant networks are essential to test the generality of these results and reveal context dependencies on changes in leaf and root traits under climate change. More specifically, cross‐site studies can test the effects of experimental duration (i.e. whether immediate responses persist over the long term), the magnitude of climate change (i.e. whether weaker or stronger treatments elicit responses in the same direction but with varying magnitudes) or differences in manipulation methods (e.g. biomass removal, root ingrowth prevention).

### Conclusion

In this study, we confirmed that fast vs slow and do‐it‐yourself vs outsourcing orthogonal trade‐offs of plants upscale to plant communities with the exception of root tissue density, that they vary with climatic conditions along mountain slopes, and that they respond to experimental climate change. Tracking the transient dynamics of these strategies, until they reach full acclimation, under simultaneous warming and drying allowed us to detect their rapid, yet not always successful acclimation. Our study highlights the importance of multiple stressors in altitudinal gradients, and that climate change effects beyond warming such as drought can lead the direction of the responses. The cascading effects between functional traits and ecosystem functioning, and the impairment of their links under climate change, show that studying the transient dynamics of functional traits can reveal early warning signals.

## Competing interests

None declared.

## Author contributions

BB, GR, WT and TM designed the research. BB, GR, AS, JR, MG, J‐CC, JP and TM conducted the fieldwork and data collection. BB, GR, CA, AS, AF, JP, EL, TM and J‐CC conducted the laboratory work. BB and RF conducted the data analysis. BB led the interpretation and writing of the manuscript with substantial support from TM. All authors contributed to the final version of the manuscript.

## Disclaimer

The New Phytologist Foundation remains neutral with regard to jurisdictional claims in maps and in any institutional affiliations.

## Supporting information


**Fig. S1** Comparison of the distribution of leaf nitrogen content at the community level.
**Fig. S2** Comparison of the leaf nitrogen content values at the species level.
**Fig. S3** Community taxonomic change over the years in AlpineWarmed communities.
**Fig. S4** Changes in the relative cover of the two species (*Patzkea paniculata* (L.) *G. H. Loos subsp. Paniculata* and *Trifolium alpinum* L.).
**Fig. S5** Community‐level functional trait values of experimental plots.
**Fig. S6** Ecosystem and microbial functions of the experimental plots.
**Notes S1** Evaluating the quality of the leaf nitrogen content measurements.
**Notes S2** Quantitative polymerase chain reaction protocol.
**Notes S3** Changes in community composition under experimental climate change.
**Table S1** Plant functional trade‐offs along temperature, moisture and soil nutrient stress gradients.
**Table S2** List of species sampled for the aboveground trait measurements.
**Table S3** ANOVA model results.
**Table S4** Averages of the experimental groups.
**Table S5** Contrast results.
**Table S6** Permutation tests for the redundancy analysis model built between functional traits as response variables and environmental variables as explanatory variables.
**Table S7** Permutation tests for the redundancy analysis model built between ecosystem function and microbial communities as explanatory variables and functional traits as response variables.
**Table S8** Loadings of the functional traits after varimax rotation on the first two axes of the principal component analysis built with the functional traits.
**Table S9** Relative contributions of the response variables to the first two axes of redundancy analysis built between ecosystem and microbial functions and functional traits.
**Table S10** Relative contributions of the explanatory variables to the first two axes of redundancy analysis built between ecosystem and microbial functions and functional traits.
Please note: Wiley is not responsible for the content or functionality of any Supporting Information supplied by the authors. Any queries (other than missing material) should be directed to the *New Phytologist* Central Office.

## Data Availability

Data are publicly available at doi: 10.17605/OSF.IO/J39UM. The code is publicly available: https://github.com/billurbektas/transalp_functional_traits.git.
