# Supplementary material for: Traits and functions of alpine plant communities respond strongly but not always sufficiently to in situ climate change
Source: New Phytol. 2025 Aug 29;249(3):1173–87. doi: 10.1111/nph.70503 (PMC12780316; doi:10.1111/nph.70503)
Supplement: Supplementary file 1 — Fig. S1 Comparison of the distribution of leaf nitrogen content at the community level. Fig. S2 Comparison of the leaf nitrogen content values at the species level. Fig. S3 Community taxonomic change over the years in AlpineWarmed communities. Fig. S4 Changes in the relative cover of the two species (Patzkea paniculata (L.) G. H. Loos subsp. Paniculata and Trifolium alpinum L.). Fig. S5 Community‐level functional trait values of experimental plots. Fig. S6 Ecosystem and microbial functions of the experimental plots. Notes S1 Evaluating the quality of the leaf nitrogen content measurements. Notes S2 Quantitative polymerase chain reaction protocol. Notes S3 Changes in community composition under experimental climate change. Table S1 Plant functional trade‐offs along temperature, moisture and soil nutrient stress gradients. Table S2 List of species sampled for the aboveground trait measurements. Table S3 ANOVA model results. Table S4 Averages of the experimental groups. Table S5 Contrast results. Table S6 Permutation tests for the redundancy analysis model built between functional traits as response variables and environmental variables as explanatory variables. Table S7 Permutation tests for the redundancy analysis model built between ecosystem function and microbial communities as explanatory variables and functional traits as response variables. Table S8 Loadings of the functional traits after varimax rotation on the first two axes of the principal component analysis built with the functional traits. Table S9 Relative contributions of the response variables to the first two axes of redundancy analysis built between ecosystem and microbial functions and functional traits. Table S10 Relative contributions of the explanatory variables to the first two axes of redundancy analysis built between ecosystem and microbial functions and functional traits. Please note: Wiley is not responsible for the content or functionality of any Supporting Information supplied by the autho [file NPH-249-1173-s001.pdf]

New Phytologist Supporting Information

Article title: Traits and functions of alpine plant communities respond strongly but not always sufficiently to *in situ* climate change

Authors: Billur Bektaş[1,2], Gemma Rutten[3], Amélie Saillard[1], Rodrigue Friaud[4], Cindy Arnoldi[1], Julien Renaud[1], Maya Guéguen[1], Arnaud Foulquier[1], Jérôme Poulenard[5], Emilie Lyautey[6], Jean-Christophe Clément[6], Wilfried Thuiller[1], Tamara Münkemüller[1]

Article acceptance date: 21 July 2025

[1]Univ. Grenoble Alpes, Univ. Savoie Mont Blanc, CNRS, LECA, F-38000, Grenoble, France
[2]Institute of Integrative Biology, Department of Environmental Systems Science, ETH Zürich, 8092, Zürich, Switzerland
[3]Institute of Plant Sciences, University of Bern, CH-3013 Bern, Switzerland
[4]Departement of Biology, École Normale Supérieure, PSL University, 75005, Paris, France
[5]Univ. Savoie Mont-Blanc, Univ. Grenoble Alpes, CNRS, EDYTEM, F-73000, Chambéry, France
[6]University Savoie Mont Blanc, INRAE, CARRTEL, 74200, Thonon-Les-Bains, France

Note S1: Evaluating the quality of the leaf nitrogen content measurements

We compared the leaf nitrogen content data to the data downloaded from TRY database. For this, we downloaded all the LNC data available in TRY database and we deleted the values if i) they derived from experimental datasets, ii) the values were not geolocalized and iii) the error risk (i.e., a TRY criterion which measures the deviation from mean trait values within species, genus, family, or the entire dataset in standard deviation) was above 4. We identified the Köppen-Geiger climatic zones of the geolocalized LNC values (Beck *et al.*, 2018) and kept the values coming only from the cold and polar (except EF - polar frost) regions. We additionally selected only for the species present in our communities. Finally, we had 1603 LNC values from 46 species (out of 81 species in our communities). Comparing the distributions of all the LNC values from TRY and the values that we measured in 2021 (Local-2021) (Fig. S1) and values per species (Fig. S2), we found that the ranges were very similar both at the trait distributions and species level. The maximum value from Local-2021 was 1% higher than the TRY values (Fig. 1) but this was due to a single species *"Campanula scheuchzeri"*. This can be due to plasticity however because there were no other individuals of this species sampled for comparison, we decided to take this value out.

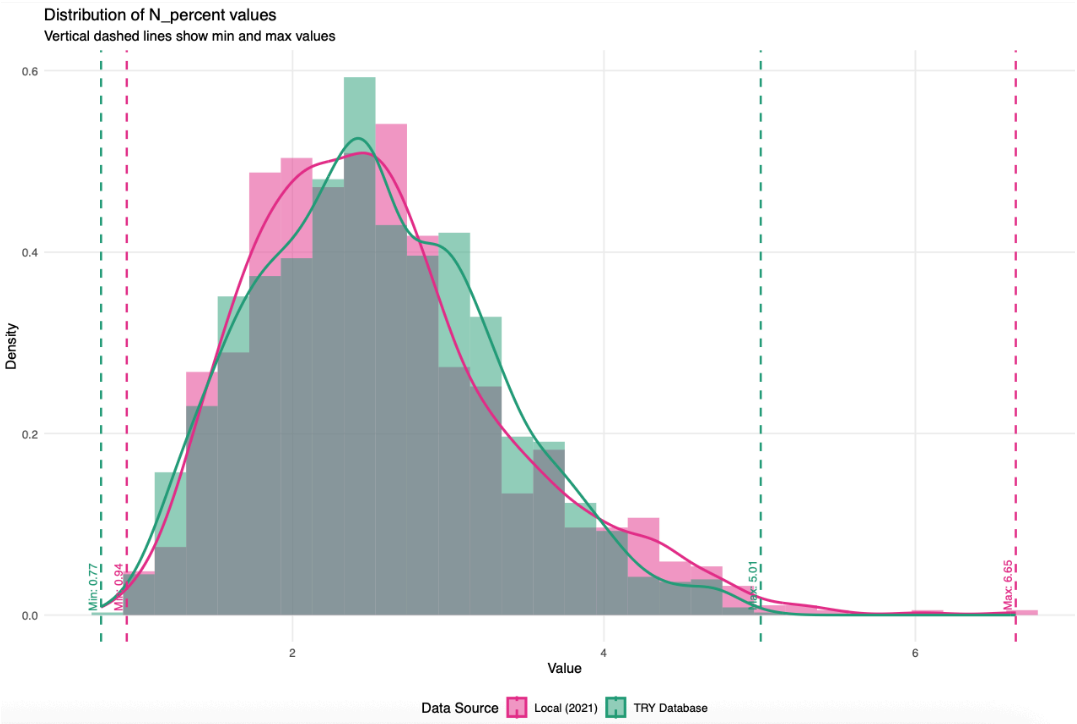

Fig. S1: Comparison of the distribution of leaf nitrogen content (LNC-%) values between *in situ* measured traits from all the experimental treatments and downloaded from TRY database to assess if the ranges of the LNC values are comparable.

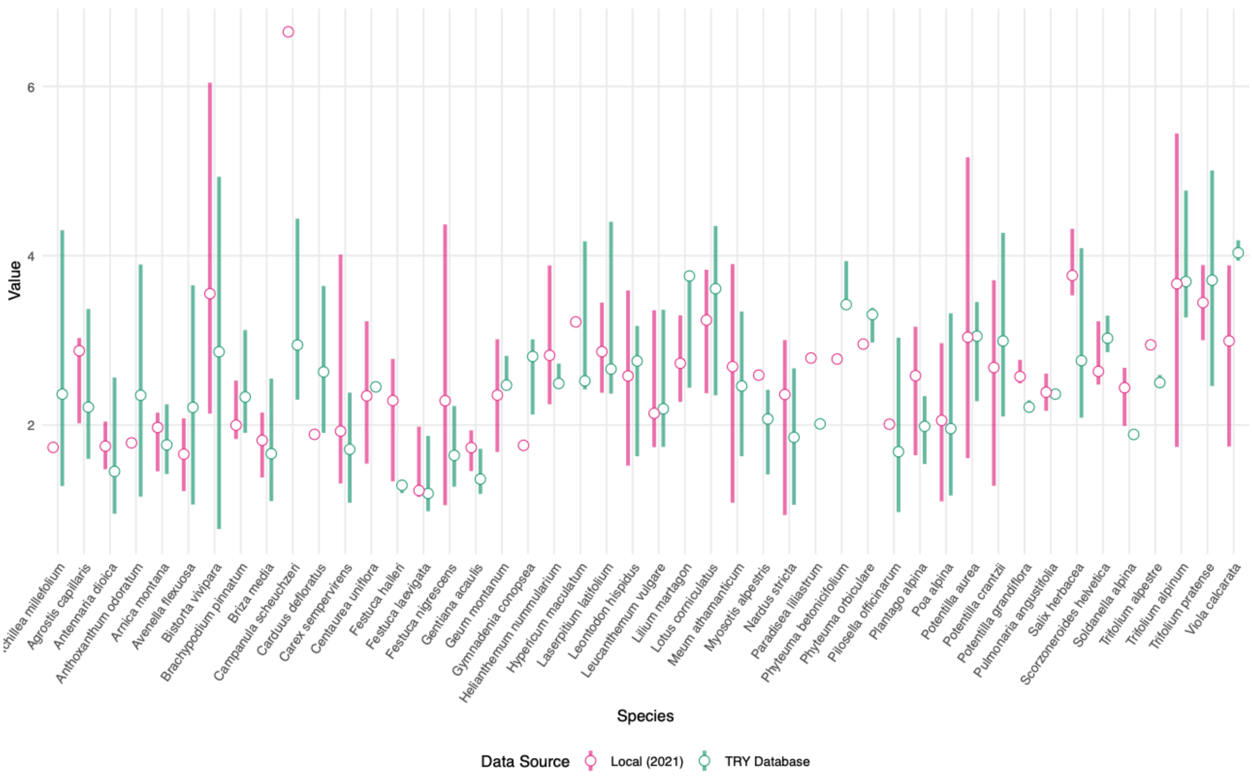

Fig. S2: Comparison of the leaf nitrogen content (LNC-%) values at species level between *in situ* measured traits from all the experimental treatments and downloaded from TRY database to assess if the ranges of the LNC values are comparable.

Note S2: qPCR protocol

Total DNA was extracted from 0.5 g of dried-ground root samples using a NucleoSpin Soil Kit following the manufacturer's instructions, and using SL1 lysis buffer and additive Enhancer SX buffer (Macherey-Nagel, Hoerdt, France). The quantity and the quality of extracted DNA were quantified and controlled using Qubit assay (Qubit 1× dsDNA HS assay kit) and agarose gel electrophoresis, respectively. The abundance of total bacteria was assessed by quantitative PCR targeting the 16S rRNA gene. Real-time PCR was carried out in triplicate on DNA extracts for each sample. Reactions were done in a final volume of 25 µl containing 1 × Takyon Master Mix (Eurogentec), 0.3 mg ml^{-1} bovine serum albumin (Sigma-Aldrich), 0.3 µM of each primer (EUB-341F: 5’-CCT ACG GGA GGC AGC AG-3’ and EUB-515R: 5’-ATT ACC GCG GCT GCT GG-3’) (López-Gutiérrez *et al.*, 2004), and 5 µl of DNA extract diluted to a 1 ng μL^{-1} concentration. Thermal cycling conditions were an initial cycle of 95°C for 3 min, 45 cycles of 95°C for 3 s and 60°C for 30 s, and a melt-curve step (55°C–95°C). A plasmid containing a single copy of 16S rRNA genes amplified from *Escherichia coli* K-12 MG1655 (Bacteria) was

diluted from 10^8 to 10^2 copies per assay and used in triplicate to produce the standard curve. Amplification efficiency was 105%.

Note S3: Changes in community composition under experimental climate change

Community composition of the AlpineWarmed communities change over the experimentals due to two processes: relative abundances of the alpine species change as some alpine species go extinct and surrounding subalpine species colonize in the AlpineWarmed communities. Over the years, we expect that AlpineWarmed communities taxonomically diverge from AlpineControl communities and converge towards SubalpineControl communities. We tested this hypothesis with 44 different whole-community transplant experiments over 22 elevational gradients across 3 continents (Bektaş *et al.*, 2024). We found that even though warmed communities follow the above-described expected trajectory, the community restructuring is not fast. Therefore, we observe colonization and extinction lags. Our experiment was one of these analyzed experiments. Below we summarize the statistical analysis that we conducted in that publication (Bektaş *et al.*, 2024) and share the results for our single experiment in France. Individual results from other experiments can also be visualized at the following web application: https://billurbektas.shinyapps.io/transplantshiny-1/

To quantify the plant community taxonomic change over time, we built principal response curves (PRC; (Van den Brink & Braak, 1999)). PRC, developed from redundancy analysis (RDA), is an ordination technique that quantifies the impact over time of several treatments on species relative abundances in experimental communities, relative to a reference community. It accounts specifically for the interaction between experimental years and treatment while controlling for the overall temporal trend in community composition. PRC allows for extracting 1) taxonomic distances between the treatments and the reference over time as canonical coefficients of the treatments and 2) species weights that quantify species' contributions to overall community taxonomic changes (Vandvik *et al.*, 2014). We built a PRC for our experiment in France using the SubalpineControl (destination control in the original publication) as the reference, with the AlpineWarmed (warmed in the original publication) and AlpineControl (origin control in the original publication) as treatments to quantify their taxonomic distance from the SubalpineControl. Canonical coefficients (Ct) from PRC axis 1 (hereafter PRC1) capture the most important taxonomic variation in differences between warmed and control communities for a given year t, quantifying AlpineWarmed communities' divergence from the AlpineControl and convergence towards the SubalpineControl. In PRCs, taxonomic distances between the AlpineControl and SubalpineControl indicate the maximum expected divergence of warmed communities from AlpineControls and their maximum expected convergence towards SubalpineControls. Moreover, species weights (i.e. constant over the years) allow us to obtain the fitted response curves for a given species and quantify the affinity of a given species with the overall trajectory of the community (i.e. the higher the species' weights, the more likely it is that the species is aligned with the pattern of community change as quantified by PRC).

Fig. S3 shows that five years after the transplantation the AlpineWarmed communities did not fully converge to the SubalpineControl communities. They are still more similar to the AlpineControl communities than to the SubalpineControl communities. Moreover, the change in

our communities is particularly slower than the other experiments worldwide (Bektaş *et al.*, 2024). The lag in community taxonomic changes are driven by the colonization lags (eg. *Patzkea paniculata (L.) G. H. Loos subsp. paniculata*, Fig. S4) and extinction lags (eg. *Trifolium alpinum L.*, Fig. S4).

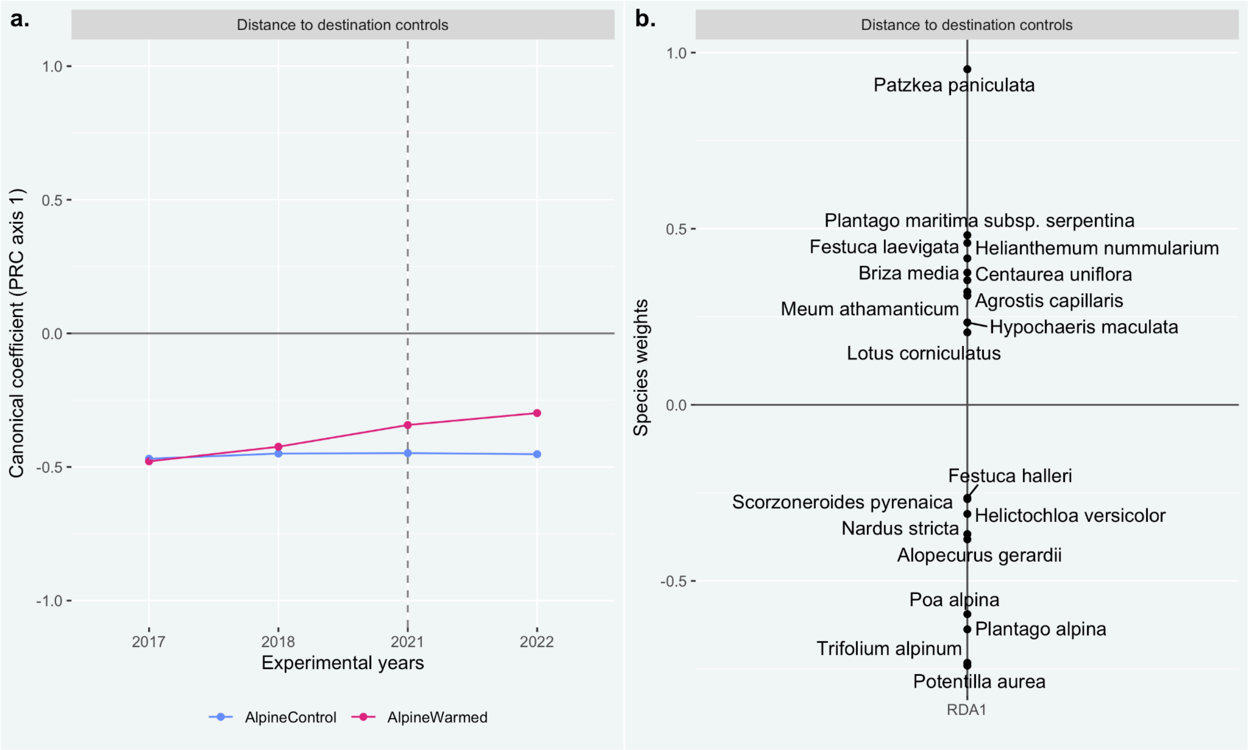

Fig. S3: Principal response curves (PRCs) were used to quantify taxonomic community change over the years in AlpineWarmed communities and at the time of leaf and root sampling (dashed line – 2021). (a) PRCs show taxonomic distances between AlpineControl and SubalpineControl communities (blue line) and between AlpineWarmed and SubalpineControl (pink line). (b) Species weights indicate the contribution of species' relative abundances to these taxonomic differences and their alignment with overall community trajectories. If a species has weights opposite in sign to the canonical treatment coefficients, it suggests an opposite response on average. For example, *Potentilla aurea L.* and *Trifolium alpinum L.* have the lowest species weights, meaning they are more abundant in AlpineControl and AlpineWarmed communities than in SubalpineControl. Similarly, *Festuca halleri All.* is also more abundant in AlpineControl and AlpineWarmed than in SubalpineControl but contributes less to taxonomic distances than *Potentilla aurea* and *Trifolium alpinum L.* In contrast, *Patzkea paniculata (L.) G. H. Loos subsp. paniculata* has the highest positive weight, indicating that it is scarcely present in AlpineControl and AlpineWarmed communities but highly abundant in SubalpineControl.

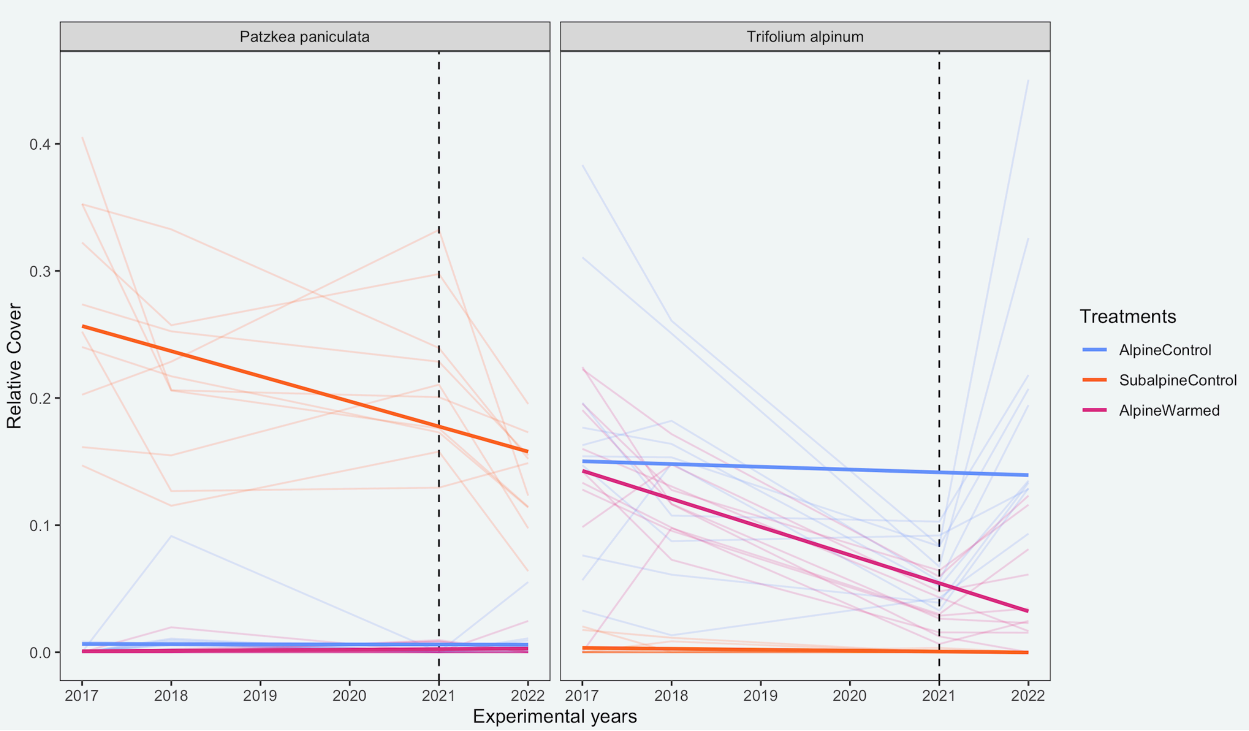

Fig. S4: Changes in the relative cover of the two species (*Patzkea paniculata (L.) G. H. Loos subsp. paniculata* and *Trifolium alpinum L.*) in the communities over the years and in the year of leaf and root sampling (dashed line - 2021).

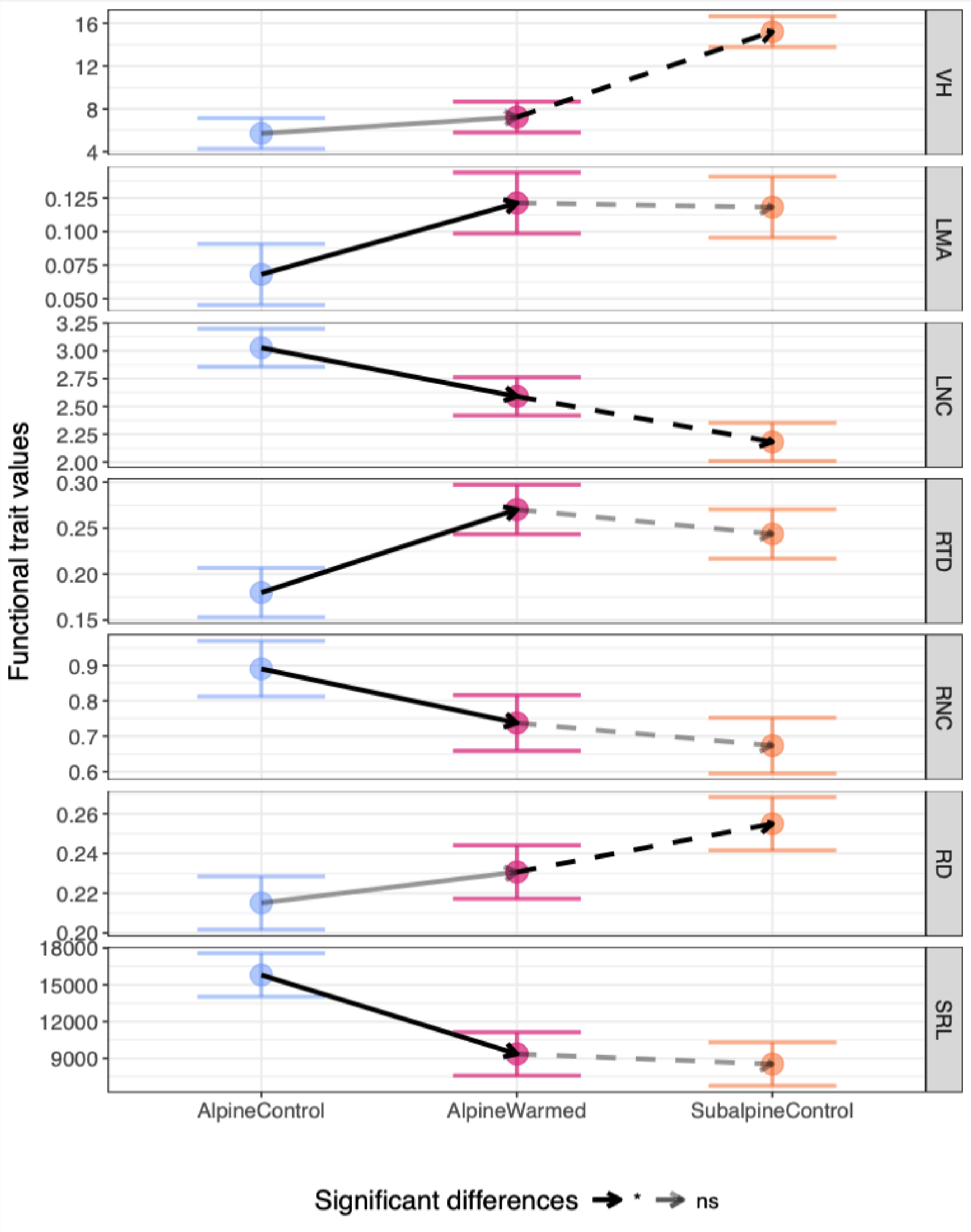

Fig. S5: Community-level functional trait values of experimental plots. The experimental climate change effect (in black solid lines) is the difference between AlpineWarmed and AlpineControl plots. The direction of the arrows indicate an increase or a decrease with the experimental

climate change effect. The acclimation lag after experimental climate change (in black dashed lines) is the difference between the AlpineWarmed and SubalpineControl plots. Non-significant differences between experimental plots are transparent. Error bars represent 95% confidence intervals. (VH: Vegetative height (cm), LNC: leaf nitrogen content (%), LMA: leaf mass per area (kg/m^2), RNC: root nitrogen content (%), SRL: specific root length(cm/g), RD: root diameter (mm), RTD: Root tissue density (g/cm^3)). (ns: non-significant)

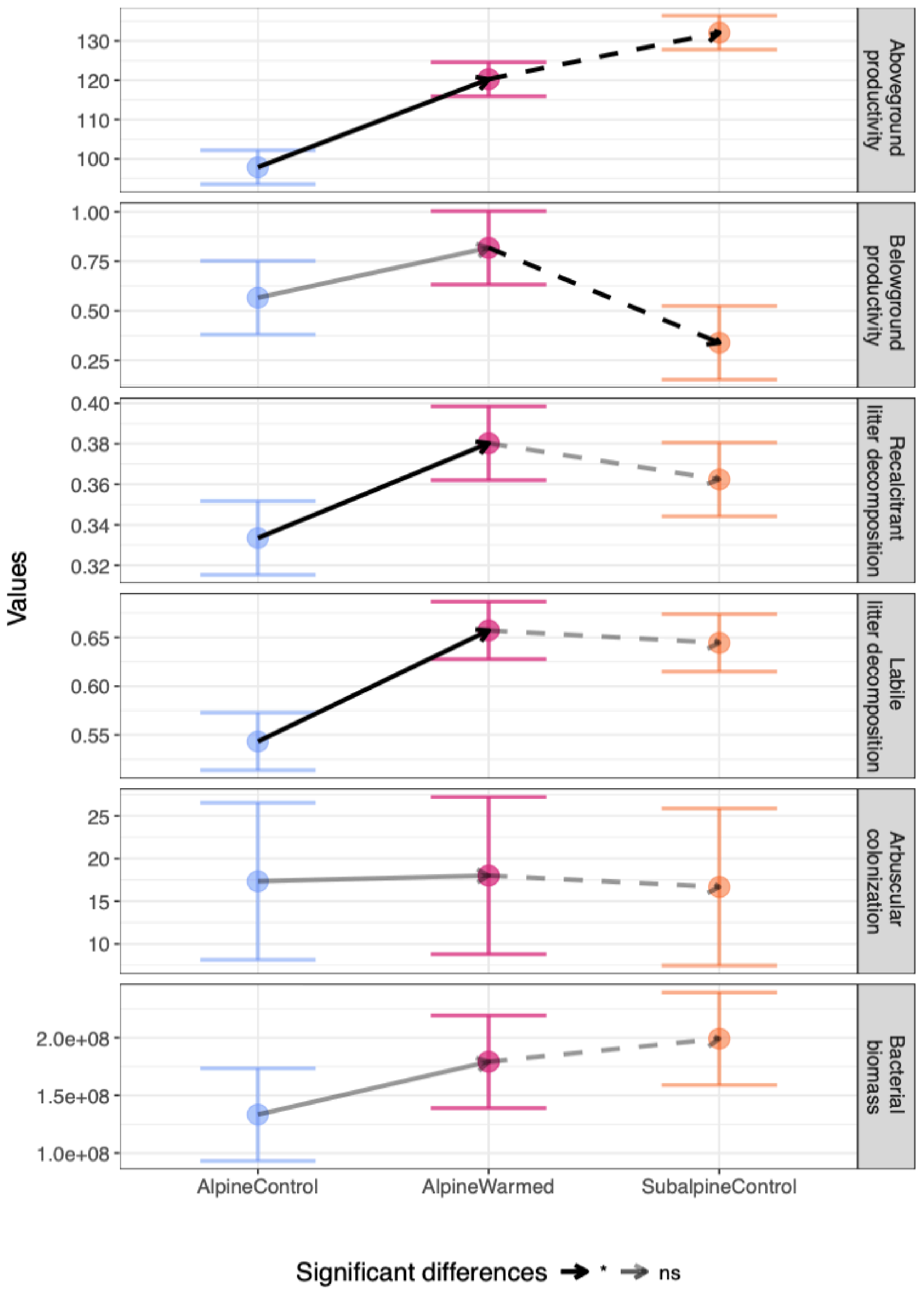

Values
Aboveground productivity
Belowground productivity
Recalcitrant litter decomposition
Labile litter decomposition
Arbuscular colonization
Bacterial biomass
130
120
110
100
1.00
0.75
0.50
0.25
0.40
0.38
0.36
0.34
0.32
0.65
0.60
0.55
25
20
15
10
2.0e+08
1.5e+08
1.0e+08
AlpineControl
AlpineWarmed
SubalpineControl
Significant differences
*
ns

Fig. S6: Ecosystem and microbial functions of the experimental plots. The experimental climate change effect (in black solid lines) is the difference between AlpineWarmed and AlpineControl plots. The direction of the arrows indicate an increase or a decrease with the experimental climate change. The acclimation lag after experimental climate change (in black dashed lines) is the difference between the AlpineWarmed and SubalpineControl plots. Non-significant differences between experimental plots are transparent. Error bars represent 95% confidence intervals. Above-ground productivity is measured as the area under the NDVI curve. NDVI values range between -1 and 1. Below-ground productivity is in g. Decomposition rates are a ratio of mass (g) loss of tea bags. Arbuscular colonization is in %. Bacterial biomass is in DNA copier per g soil. (ns: non-significant)

Table S1: Plant functional trade-offs along temperature, moisture and soil nutrient stress gradients. Arrows indicate the increasing or decreasing trend for each trait as a response to different abiotic drivers. Blue arrows show hypothesized trends with an overall consensus on existing studies. Red arrows show hypothesized trends but with some contrasting evidence. (LMA: leaf mass per area, LNC: leaf nitrogen content, RNC: root nitrogen content, SRL: specific root length, RD: root diameter). References supporting our hypotheses are indicated in parentheses. References showing contrasting results to our hypotheses are also indicated after “but see” notation.

		**Increasing temperature**	**Decreasing moisture**	**Decreasing soil nutrients**
**Fast vs. slow trade-off**	**LMA**	↓ (Simpson *et al.*, 2016; Kemppinen *et al.*, 2021; Joswig *et al.*, 2022) **but see** (Wright *et al.*, 2005)	↑ (Simpson *et al.*, 2016; Onoda *et al.*, 2017; Blumenthal *et al.*, 2020; Kemppinen *et al.*, 2021; Joswig *et al.*, 2022)	↑ (Simpson *et al.*, 2016; Martinez-Almoyna *et al.*, 2020; Weil *et al.*, 2021; Joswig *et al.*, 2022)
	**LNC**	↑ (Wright *et al.*, 2005; Kemppinen *et al.*, 2021; Joswig *et al.*, 2022)	↓ (Onoda *et al.*, 2017; Kemppinen *et al.*, 2021; Joswig *et al.*, 2022) **but see** (Wright *et al.*, 2005)	↓ (Martinez-Almoyna *et al.*, 2020; Weil *et al.*, 2021; Joswig *et al.*, 2022)
	**RNC**	↑ (Scheurwater *et al.*, 2002; Freschet *et al.*, 2021) **but see** (Laughlin *et al.*, 2021)	↓ (Scheurwater *et al.*, 2002; Freschet *et al.*, 2021) **but see** (Laughlin *et al.*, 2021)	↓ (Freschet *et al.*, 2018, 2021)
**Outsourcing vs. do-it-yourself trade-off**	**SRL**	↑ **But see** (Simpson *et al.*, 2016; Laughlin *et al.*, 2021)	↓ (Augé *et al.*, 2015; Simpson *et al.*, 2016; Laughlin *et al.*, 2021)	↑ (Freschet *et al.*, 2021)
	**RD**	↓ **But see** (Laughlin *et al.*, 2021)	↑ But see (Laughlin *et al.*, 2021)	↓

Table S2: List of species sampled for the aboveground trait measurements. The information on the mycorrhizal associations were compiled from GRoot database (Guerrero-Ramírez *et al.*, 2021) and the species were matched at the species level and not at subspecies or variety level. AM: Arbuscular mycorrhizal, NM: Non-mycorrhizal, AM-NM: both possible, EcM: Ecto-mycorrhizal, OM: Orchid mycorrhizal

Species	Growth form	Mycorrhizal association	Experimental group
Achillea millefolium	forb	AM	SubalpineControl
Agrostis capillaris	graminoid	AM	AlpineWarmed, SubalpineControl
Alchemilla fissa	forb	AM	AlpineControl, SubalpineControl
Alchemilla hybrida	forb	AM	SubalpineControl
Alopecurus aequalis	graminoid	AM	AlpineControl
Alopecurus gerardi	graminoid	AM	AlpineControl, AlpineWarmed
Antennaria dioica	forb	AM	AlpineWarmed, SubalpineControl
Anthoxanthum odoratum	graminoid	AM	AlpineControl, AlpineWarmed, SubalpineControl
Anthyllis vulneraria	forb	AM	SubalpineControl
Arnica montana	forb	AM	SubalpineControl
Aster alpinus	forb	AM	SubalpineControl
Avenella flexuosa	graminoid	AM	AlpineWarmed, SubalpineControl
Bistorta vivipara	forb	EcM	AlpineControl, AlpineWarmed, SubalpineControl
Brachypodium pinnatum	graminoid	AM	SubalpineControl
Briza media	graminoid	AM	AlpineWarmed, SubalpineControl
Bupleurum falcatum	forb	AM	SubalpineControl
Campanula scheuchzeri	forb	AM	AlpineControl, AlpineWarmed
Carduus defloratus	forb	AM	SubalpineControl
Carex foetida	graminoid	NM-AM	AlpineControl
Carex halleriana subsp. halleriana	graminoid	NM-AM	AlpineControl
Carex sempervirens	graminoid	NM-AM	AlpineControl, AlpineWarmed, SubalpineControl
Carex sempervirens subsp. sempervirens	graminoid	NM-AM	AlpineControl
Carlina acaulis subsp. caulescens	forb	AM	AlpineWarmed, SubalpineControl
Centaurea uniflora	forb	AM	SubalpineControl

Euphrasia salisburgensis	forb	NM	SubalpineControl
Festuca halleri	graminoid	AM	AlpineWarmed
Festuca laevigata	graminoid	AM	SubalpineControl
Festuca nigrescens	graminoid	AM	AlpineControl, AlpineWarmed, SubalpineControl
Galium pumilum	forb	NM-AM	SubalpineControl
Gentiana acaulis	forb	AM	AlpineWarmed, SubalpineControl
Geum montanum	forb	AM	AlpineControl, AlpineWarmed
Gymnadenia conopsea	forb	OM	SubalpineControl
Helianthemum nummularium	forb	EcM	SubalpineControl
Helictochloa versicolor	graminoid	AM	AlpineControl, AlpineWarmed, SubalpineControl
Hieracium murorum	forb	AM	SubalpineControl
Hieracium piliferum	forb	AM	AlpineControl
Homogyne alpina	forb	AM	AlpineControl
Hypericum maculatum	forb	AM	AlpineWarmed
Hypericum richeri	forb	AM	SubalpineControl
Hypochaeris maculata	forb	AM	AlpineWarmed, SubalpineControl
Hypochaeris radicata	forb	AM	SubalpineControl
Laserpitium latifolium	forb	AM	SubalpineControl
Leontodon hispidus	forb	AM	AlpineControl, AlpineWarmed, SubalpineControl
Leucanthemum adustum	forb	AM	SubalpineControl
Leucanthemum vulgare	forb	AM	SubalpineControl
Lilium martagon	forb	AM	SubalpineControl
Lotus corniculatus	forb	AM	SubalpineControl
Luzula pediformis	graminoid	NM	AlpineWarmed
Meum athamanticum	forb	AM	AlpineWarmed, SubalpineControl
Myosotis alpestris	forb	NM-AM	AlpineControl
Nardus stricta	graminoid	AM	AlpineControl, AlpineWarmed, SubalpineControl
Paradisea liliastrum	forb	AM	SubalpineControl
Patzkea paniculata subsp. paniculata	graminoid	AM	SubalpineControl, AlpineWarmed
Phleum alpinum	graminoid	AM	AlpineControl, AlpineWarmed

Phyteuma betonicifolium	forb	AM	SubalpineControl
Phyteuma orbiculare	forb	AM	SubalpineControl
Pilosella officinarum	forb	AM	SubalpineControl
Plantago alpina	forb	AM	AlpineControl, AlpineWarmed
Plantago maritima subsp. serpentina	forb	AM	AlpineWarmed, SubalpineControl
Plantago sempervirens	forb	AM	AlpineControl, SubalpineControl
Poa alpina	graminoid	AM	AlpineControl, AlpineWarmed
Poa alpina var. alpina	graminoid	AM	AlpineControl
Polygala vulgaris	forb	AM	SubalpineControl
Potentilla aurea	forb	NM-AM	AlpineControl, AlpineWarmed, SubalpineControl
Potentilla crantzii	forb	NM-AM	AlpineControl, AlpineWarmed, SubalpineControl
Potentilla grandiflora	forb	NM-AM	AlpineControl, AlpineWarmed, SubalpineControl
Pulmonaria angustifolia	forb	AM	SubalpineControl
Ranunculus kuepferi	forb	AM	AlpineControl, AlpineWarmed
Sagina glabra	forb	NM	AlpineWarmed
Salix herbacea	shrub	EcM-AM	AlpineControl
Scorzonera austriaca	forb	AM	AlpineControl
Scorzonera humilis	forb	AM	AlpineControl
Scorzoneroides pyrenaica var. helvetica	forb	AM	AlpineControl, AlpineWarmed, SubalpineControl
Senecio doronicum subsp. doronicum	forb	AM	SubalpineControl
Soldanella alpina	forb	AM	AlpineControl
Trifolium alpestre	legume	AM	SubalpineControl
Trifolium alpinum	legume	AM	AlpineControl, AlpineWarmed
Trifolium montanum	legume	AM	SubalpineControl
Trifolium pratense	legume	AM	SubalpineControl
Trisetum flavescens subsp. flavescens	graminoid	AM	SubalpineControl
Veronica allionii	forb	NM-AM	AlpineWarmed, SubalpineControl
Viola calcarata	forb	AM	AlpineControl, AlpineWarmed, SubalpineControl

Table S3: ANOVA model results

Variable	r.squared	adj.r.squared	sigma	statistic	p.value	df	logLik	AIC	BIC	deviance	df.residual	nobs
LMA	0.4312633358	0.389134694	0.7815787267	10.23682031	0.000491248861	2	-33.59456637	75.18913275	80.79392227	16.49336326	27	30
LNC	0.6546184164	0.6290345954	0.6090692938	25.5872028	5.85E-07	2	-26.11305121	60.22610243	65.83089196	10.01606592	27	30
RNC	0.3829112435	0.3372009652	0.8055821661	8.376917797	0.001478063366	2	-34.502046	77.00409201	82.60888153	17.52199091	27	30
RTD	0.5798003741	0.5486744759	0.66110784	18.62758691	8.25E-06	2	-28.57259909	65.14519818	70.74998771	11.80071655	27	30
SRL	0.5538637057	0.5208165727	0.6852317242	16.75981112	1.85E-05	2	-29.64780183	67.29560365	72.90039318	12.67764793	27	30
RD	0.4120813034	0.3685317703	0.7902537795	9.462358703	0.0007687465261	2	-33.92571391	75.85142781	81.45621734	16.86152797	27	30
VH	0.7982088476	0.7832613549	0.465551979	53.4008519	4.13E-10	2	-18.05180249	44.10360497	49.7083945	5.851943419	27	30
Aboveground productivity	0.8246498243	0.8116609224	0.4339805037	63.48880225	6.20E-11	2	-15.94507821	39.89015642	45.49494595	5.085155094	27	30
Belowground productivity	0.4495886802	0.4088174713	0.7656678212	11.02711184	0.0003157034446	2	-32.97754255	73.9550851	79.55987463	15.82867474	27	30
Labile litter decomposition	0.5626681574	0.5302732061	0.6867883088	17.36900766	1.42E-05	2	-29.71587308	67.43174615	73.03653568	12.73531089	27	30
Recalcitrant litter decomposition	0.34921327	0.3010068456	0.8360581047	7.24412304	0.003029858164	2	-35.61603331	79.23206662	84.83685614	18.87281517	27	30
Bacterial biomass	0.1697498521	0.1082498412	0.904993051	2.760159705	0.0811560651	2	-37.9929078	83.98581572	89.59060524	22.11333542	27	30

			3		1		6					
Arbuscular colonization	0.00162999185	-0.07232334209	1.017562761	0.02204081633	0.978217889	2	-41.5100578	91.0201156	96.62490513	27.95671726	27	30
Moisture	0.9160505671	0.9098320906	0.3002797187	147.3110922	2.98E-15	2	-4.896522967	17.79304593	23.39783546	2.434533555	27	30
Nitrate concentration	0.1118083329	0.04601635756	0.9767208621	1.699422039	0.2017624644	2	-40.28111695	88.5622339	94.16702343	25.75755835	27	30
Total P concentration	0.4940774788	0.4566017364	0.737155522	13.18392774	0.0001011892016	2	-31.8390566	71.6781132	77.28290272	14.67175312	27	30

Table S4: Averages of the experimental groups

**group**	**variable**	**estimate**	**std.error**	**df**	**statistic**	**p.value**	**lower.CL**	**upper.CL**
AlpineControl	LMA	-0.9108172859	0.2471568947	27	-3.685178546	0.001011557887	-1.417941345	-0.403693227
AlpineWarmed	LMA	0.5114509544	0.2471568947	27	2.069337192	0.04820894771	0.004326895459	1.018575013
SubalpineControl	LMA	0.3993663315	0.2471568947	27	1.615841355	0.1177550634	-0.1077577274	0.9064903905
AlpineControl	LNC	0.9605884456	0.1926046221	27	4.98735926	3.15E-05	0.5653964043	1.355780487
AlpineWarmed	LNC	0.02680446829	0.1926046221	27	0.1391683543	0.8903499913	-0.368387573	0.4219965096
SubalpineControl	LNC	-0.9873929138	0.1926046221	27	-5.126527614	2.17E-05	-1.382584955	-0.5922008726
AlpineControl	RNC	0.7949979631	0.2547474487	27	3.120729833	0.004264961677	0.2722993738	1.317696552
AlpineWarmed	RNC	-0.2199753657	0.2547474487	27	-0.8635037046	0.3954667674	-0.742673955	0.3027232236
SubalpineControl	RNC	-0.6389139971	0.2547474487	27	-2.508029031	0.0184531119	-1.161612586	-0.1162154078
AlpineControl	RTD	-1.000397651	0.2090606553	27	-4.785202884	5.43E-05	-1.429354683	-0.5714406185
AlpineWarmed	RTD	0.7357323409	0.2090606553	27	3.519229095	0.001554634564	0.3067753084	1.164689373

SubalpineControl	RTD	0.2940725668	0.2090606553	27	1.406637544	0.1709413589	-0.1348844656	0.7230295992
AlpineControl	SRL	0.9917808688	0.2166892973	27	4.576972102	9.49E-05	0.5471711559	1.436390582
AlpineWarmed	SRL	-0.4009948039	0.2166892973	27	-1.85055196	0.07520507756	-0.8456045168	0.043614909
SubalpineControl	SRL	-0.656428961	0.2166892973	27	-3.029355714	0.005346083291	-1.101038674	-0.2118192482
AlpineControl	RD	-0.6903237574	0.2499001873	27	-2.762397919	0.01019898024	-1.203076588	-0.177570927
AlpineWarmed	RD	-0.06454130302	0.2499001873	27	-0.2582683259	0.7981584047	-0.5772941334	0.4482115273
SubalpineControl	RD	0.838738956	0.2499001873	27	3.356295828	0.002358786754	0.3259861257	1.351491786
AlpineControl	VH	-0.8311523275	0.1472204623	27	-5.645630469	5.41E-06	-1.133223765	-0.5290808903
AlpineWarmed	VH	-0.3839813298	0.1472204623	27	-2.60820625	0.01465196399	-0.686052767	-0.08190989265
SubalpineControl	VH	1.215133657	0.1472204623	27	8.253836718	7.33E-09	0.9130622201	1.517205094
AlpineControl	Aboveground productivity	-1.20298899	0.1372366852	27	-8.765797486	2.21E-09	-1.484575409	-0.9214025713
AlpineWarmed	Aboveground productivity	0.2692967182	0.1372366852	27	1.962279385	0.06011245481	-0.0122897004	0.5508831368
SubalpineControl	Aboveground productivity	0.9336922717	0.1372366852	27	6.803518101	2.63E-07	0.6521058531	1.21527869
AlpineControl	Belowground productivity	0.08703613505	0.2421254246	27	0.3594671447	0.7220418971	-0.4097642	0.5838364701
AlpineWarmed	Belowground productivity	0.7098763555	0.2421254246	27	2.931853838	0.006785792876	0.2130760205	1.206676691
SubalpineControl	Belowground productivity	-0.8854583229	0.2421254246	27	-3.657023315	0.001088426136	-1.382258658	-0.3886579878
AlpineControl	Labile litter decomposition	-1.076620563	0.2171815326	27	-4.957238077	3.42E-05	-1.522240259	-0.6310008668
AlpineWarmed	Labile litter decomposition	0.5676296111	0.2171815326	27	2.613618222	0.01446894178	0.1220099149	1.013249307
SubalpineControl	Labile litter decomposition	0.4013288956	0.2171815326	27	1.847896047	0.07560050943	-0.04429080064	0.8469485919
AlpineControl	Recalcitrant litter decomposition	-0.767597289	0.2643847867	27	-2.903333806	0.007272112603	-1.310070062	-0.2251245155

AlpineWarmed	Recalcitrant litter decomposition	0.637678622	0.2643847867	27	2.411933871	0.02293717283	0.09520584854	1.180151395
SubalpineControl	Recalcitrant litter decomposition	0.129918667	0.2643847867	27	0.4913999349	0.6271146314	-0.4125541065	0.6723914404
AlpineControl	Bacterial biomass	-0.497958764	0.2861839309	27	-1.739995542	0.0932485062	-1.085159687	0.08924215873
AlpineWarmed	Bacterial biomass	0.09417986749	0.2861839309	27	0.3290885942	0.7446278853	-0.4930210552	0.6813807902
SubalpineControl	Bacterial biomass	0.4424787751	0.2861839309	27	1.546134242	0.1337141626	-0.1447221476	1.029679698
AlpineControl	Arbuscular colonization	-0.001647316486	0.3217815987	27	-0.005119361993	0.9959529963	-0.6618886203	0.6585939873
AlpineWarmed	Arbuscular colonization	0.04612486162	0.3217815987	27	0.1433421358	0.8870844321	-0.6141164422	0.7063661654
SubalpineControl	Arbuscular colonization	-0.04941949459	0.3217815987	27	-0.1535808598	0.8790823627	-0.7096607984	0.6108218092
AlpineControl	Moisture	1.327436029	0.09495678462	27	13.97937003	6.99E-14	1.132600801	1.522271257
AlpineWarmed	Moisture	-0.7456235336	0.09495678462	27	-7.85224075	1.92E-08	-0.9404587621	-0.5507883052
SubalpineControl	Moisture	-0.5818124953	0.09495678462	27	-6.127129279	1.51E-06	-0.7766477237	-0.3869772669
AlpineControl	Nitrate concentration	-0.07639398646	0.3088662562	27	-0.2473367839	0.8065187538	-0.7101351965	0.5573472236
AlpineWarmed	Nitrate concentration	0.4353683062	0.3088662562	27	1.40956902	0.1700820612	-0.1983729039	1.069109516
SubalpineControl	Nitrate concentration	-0.3589743197	0.3088662562	27	-1.162232236	0.2553118732	-0.9927155298	0.2747668904
AlpineControl	Total P concentration	0.9437126116	0.2331090439	27	4.048374082	0.0003893623561	0.4654123616	1.422012862
AlpineWarmed	Total P concentration	-0.2517035763	0.2331090439	27	-1.079767529	0.2897971603	-0.7300038263	0.2265966737
SubalpineControl	Total P concentration	-0.6920090352	0.2331090439	27	-2.968606553	0.00620447889	-1.170309285	-0.2137087853

Table S5: Contrast results

**contrast**	**variable**	**estimate**	**std.error**	**df**	**statistic**	**adj.p.value**	lower.CL	upper.CL

Warming effect	LMA	1.42226824	0.3495326325	27	4.069057101	0.0007156810053	0.6066204032	2.237916077
Warming lag	LMA	-0.1120846228	0.3495326325	27	-0.3206699816	0.9279659716	-0.9277324599	0.7035632143
Warming effect	LNC	-0.9337839773	0.2723840688	27	-3.428188666	0.003762710574	-1.569402675	-0.29816528
Warming lag	LNC	-1.014197382	0.2723840688	27	-3.723409327	0.001766602159	-1.649816079	-0.3785786848
Warming effect	RNC	-1.014973329	0.360267297	27	-2.817278552	0.01679281403	-1.855670918	-0.1742757395
Warming lag	RNC	-0.4189386313	0.360267297	27	-1.16285501	0.4121741711	-1.259636221	0.421758958
Warming effect	RTD	1.736129992	0.2956564141	27	5.872120166	5.85E-06	1.046204382	2.426055602
Warming lag	RTD	-0.4416597741	0.2956564141	27	-1.493827811	0.2491718607	-1.131585384	0.248265836
Warming effect	SRL	-1.392775673	0.3064449431	27	-4.544945851	0.0002021395616	-2.10787673	-0.6776746155
Warming lag	SRL	-0.2554341572	0.3064449431	27	-0.8335401282	0.6200024142	-0.9705352144	0.4596669
Warming effect	RD	0.6257824544	0.3534122341	27	1.770687016	0.1538547754	-0.1989185822	1.450483491
Warming lag	RD	0.9032802591	0.3534122341	27	2.555882824	0.03065246286	0.07857922248	1.727981296
Warming effect	VH	0.4471709977	0.2082011744	27	2.147783263	0.07385169399	-0.03867444793	0.9330164432
Warming lag	VH	1.599114987	0.2082011744	27	7.68062424	5.80E-08	1.113269541	2.084960433
Warming effect	Aboveground productivity	1.472285708	0.1940819814	27	7.585895905	7.31E-08	1.019387942	1.925183474
Warming lag	Aboveground productivity	0.6643955535	0.1940819814	27	3.423272725	0.003809803666	0.2114977876	1.117293319
Warming effect	Belowground productivity	0.6228402205	0.3424170593	27	1.818952075	0.1407241524	-0.1762031529	1.421883594
Warming lag	Belowground productivity	-1.595334678	0.3424170593	27	-4.659039715	0.0001489915213	-2.394378052	-0.796291305
Warming effect	Labile litter decomposition	1.644250174	0.3071410689	27	5.353403828	2.32E-05	0.9275246806	2.360975668

Warming lag	Labile litter decomposition	-0.1663007155	0.3071410689	27	-0.5414473424	0.8113241794	-0.8830262091	0.550424778
Warming effect	Recalcitrant litter decomposition	1.405275911	0.373896551	27	3.758461818	0.001613197581	0.5327739332	2.277777889
Warming lag	Recalcitrant litter decomposition	-0.507759955	0.373896551	27	-1.35802257	0.3094861135	-1.380261933	0.3647420227
Warming effect	Bacterial biomass	0.5921386315	0.4047251964	27	1.463063424	0.2620248433	-0.3523031755	1.536580438
Warming lag	Bacterial biomass	0.3482989076	0.4047251964	27	0.8605812307	0.6019143958	-0.5961428993	1.292740715
Warming effect	Arbuscular colonization	0.0477721781	0.455067901	27	0.1049781318	0.9919412622	-1.014146266	1.109690622
Warming lag	Arbuscular colonization	-0.0955443562	0.455067901	27	-0.2099562637	0.9682648225	-1.1574628	0.9663740875
Warming effect	Moisture	-2.073059563	0.1342891726	27	-15.43728003	1.29E-14	-2.38642851	-1.759690615
Warming lag	Moisture	0.1638110384	0.1342891726	27	1.219838019	0.380305329	-0.1495579093	0.477179986
Warming effect	Nitrate concentration	0.5117622926	0.4368028485	27	1.17160933	0.4071817521	-0.507533949	1.531058534
Warming lag	Nitrate concentration	-0.7943426259	0.4368028485	27	-1.818538108	0.1408327391	-1.813638867	0.2249536157
Warming effect	Total P concentration	-1.195416188	0.3296659714	27	-3.626143708	0.002270542308	-1.964704407	-0.4261279683
Warming lag	Total P concentration	-0.4403054589	0.3296659714	27	-1.335610882	0.3203305196	-1.209593678	0.3289827606

Table S6: Permutation tests for the RDA model built between functional traits as response variables and environmental variables as explanatory variables. (Fig. 5 in main text)

	Df	Variance	F	Pr(>F)
Model	4	3.21	4.72	0
RDA1	1	2.66	15.63	0
RDA2	1	0.42	2.46	0.24
RDA3	1	0.12	0.68	0.92

RDA4	1	0.02	0.09	1
Residual	15	2.55	NA	NA

Table S7: Permutation tests for the RDA model built between ecosystem function and microbial communities as explanatory variables and functional traits as response variables (Fig. 7 in main text).

	Df	Variance	F	Pr(>F)
Model	15	3.29	1.93	0
RDA1	1	1.65	24.96	0
RDA2	1	0.87	13.17	0.2
RDA3	1	0.33	5.04	0.99
RDA4	1	0.32	4.77	0.99
RDA5	1	0.11	1.67	1
Residual	24	1.59	NA	NA

Table S8: Loadings of the functional traits after varimax rotation on the first two axes of the principal component analysis built with the functional traits (VH: Vegetative height, LNC: leaf nitrogen content, LMA: leaf mass per area, RNC: root nitrogen content, SRL: specific root length, RD: root diameter, RTD: Root tissue density) The first four highest loadings are highlighted in bold.

Functional trait	RC1	RC2
LMA	0.11	**0.92**
LNC	-0.41	**-0.81**
RNC	-0.44	**-0.64**
RTD	**0.89**	0.25
SRL	**-0.95**	-0.29
RD	**0.88**	0.29
VH	**0.69**	**0.66**

Table S9: Relative contributions of the response variables to the first two axes of RDA (Fig. 7 - main text) in percentages

Variable	RDA1	RDA2
Above-ground productivity	40.14	8.53

Arbuscule colonization	0.19	1.27
Below-ground productivity	14.41	57.67
Bacterial biomass	20.07	9.07
Labile litter decomposition rate	25.19	23.46

Table S10: Relative contributions of the explanatory variables to the first two axes of RDA (Fig. 7 - main text) in percentages

Variable	RDA1	RDA2
LMA	6.57	6.21
LMA:eCC	0.09	10.9
LNC	13.76	0.5
LNC:eCC	0.83	0.94
RD	15.46	0.03
RD:eCC	0.67	7.45
RNC	8.68	10.5
RNC:eCC	0.18	3.66
RTD	11.88	9.86
RTD:eCC	0.07	10.19
SRL	18.54	3.84
SRL:eCC	0.47	0.59
VH	22.12	1.37
VH:eCC	0.1	13.85
eCC	0.59	20.11

References

Augé RM, Toler HD, Saxton AM. **2015**. Arbuscular mycorrhizal symbiosis alters stomatal conductance of host plants more under drought than under amply watered conditions: a meta-analysis. *Mycorrhiza* **25**: 13–24.

Beck HE, Zimmermann NE, McVicar TR, Vergopolan N, Berg A, Wood EF. **2018**. Present and future Köppen-Geiger climate classification maps at 1-km resolution. *Scientific data* **5**: 180214.

Bektaş B, Chisholm C, Egelkraut D, Lynn J, Block S, Deola T, Dommanget F, Enquist BJ, Goldberg DE, Haider S, *et al.* 2024. Colonization and extinction lags drive non-linear

responses to warming in mountain plant communities across the Northern Hemisphere. *Ecography*.

Blumenthal DM, Mueller KE, Kray JA, Ocheltree TW, Augustine DJ, Wilcox KR. **2020**. Traits link drought resistance with herbivore defence and plant economics in semi-arid grasslands: The central roles of phenology and leaf dry matter content. *The Journal of ecology* **108**: 2336–2351.

Freschet GT, Pagès L, Iversen CM, Comas LH, Rewald B, Roumet C, Klimešová J, Zadworny M, Poorter H, Postma JA, *et al.* 2021. A starting guide to root ecology: strengthening ecological concepts and standardising root classification, sampling, processing and trait measurements. *The New phytologist* **232**: 973–1122.

Freschet GT, Violle C, Bourget MY, Scherer-Lorenzen M, Fort F. **2018**. Allocation, morphology, physiology, architecture: the multiple facets of plant above- and below-ground responses to resource stress. *The New phytologist* **219**: 1338–1352.

Guerrero-Ramírez NR, Mommer L, Freschet GT, Iversen CM, McCormack ML, Kattge J, Poorter H, Plas F, Bergmann J, Kuyper TW, *et al.* 2021. Global root traits (GRooT) database. *Global ecology and biogeography: a journal of macroecology* **30**: 25–37.

Joswig JS, Wirth C, Schuman MC, Kattge J, Reu B, Wright IJ, Sippel SD, Rüger N, Richter R, Schaepman ME, *et al.* 2022. Climatic and soil factors explain the two-dimensional spectrum of global plant trait variation. *Nature ecology & evolution* **6**: 36–50.

Kemppinen J, Niittynen P, le Roux PC, Momberg M, Happonen K, Aalto J, Rautakoski H, Enquist BJ, Vandvik V, Halbritter AH, *et al.* 2021. Consistent trait–environment relationships within and across tundra plant communities. *Nature Ecology & Evolution* **5**: 458–467.

Laughlin DC, Mommer L, Sabatini FM, Bruelheide H, Kuyper TW, McCormack ML, Bergmann J, Freschet GT, Guerrero-Ramírez NR, Iversen CM, *et al.* 2021. Root traits explain plant species distributions along climatic gradients yet challenge the nature of ecological trade-offs. *Nature ecology & evolution* **5**: 1123–1134.

López-Gutiérrez JC, Henry S, Hallet S, Martin-Laurent F, Catroux G, Philippot L. **2004**. Quantification of a novel group of nitrate-reducing bacteria in the environment by real-time PCR. *Journal of microbiological methods* **57**: 399–407.

Martinez-Almoyna C, Piton G, Abdulhak S, Boulangeat L, Choler P, Delahaye T, Dentant C, Foulquier A, Poulenard J, Noble V, *et al.* 2020. Climate, soil resources and microbial activity shape the distributions of mountain plants based on their functional traits. *Ecography* **43**: 1550–1559.

Onoda Y, Wright IJ, Evans JR, Hikosaka K, Kitajima K, Niinemets Ü, Poorter H, Tosens T, Westoby M. **2017**. Physiological and structural tradeoffs underlying the leaf economics spectrum. *The New phytologist* **214**: 1447–1463.

Scheurwater I, Koren M, Lambers H, Atkin OK. **2002**. The contribution of roots and shoots to whole plant nitrate reduction in fast- and slow-growing grass species. *Journal of experimental botany* **53**: 1635–1642.

Simpson AH, Richardson SJ, Laughlin DC. **2016**. Soil-climate interactions explain variation in

foliar, stem, root and reproductive traits across temperate forests. *Global ecology and biogeography: a journal of macroecology* **25**: 964–978.

Van den Brink PJ, Braak CJFT. **1999**. Principal response curves: Analysis of time-dependent multivariate responses of biological community to stress. *Environmental toxicology and chemistry / SETAC* **18**: 138–148.

Vandvik V, Måren IE, Ndangalasi HJ, Taplin J, Mbago F, Lovett JC. **2014**. Back to Africa: monitoring post-hydropower restoration to facilitate reintroduction of an extinct-in-the-wild amphibian. *Ecosphere* **5**: 1–16.

Weil S-S, Martinez-Almoyna C, Piton G, Renaud J, Boulangeat L, Foulquier A, Saillard A, Choler P, Poulenard J, Münkemüller T, *et al.* 2021. Strong links between plant traits and microbial activities but different abiotic drivers in mountain grasslands. *Journal of biogeography* **48**: 2755–2770.

Wright IJ, Reich PB, Cornelissen JHC, Falster DS, Groom PK, Hikosaka K, Lee W, Lusk CH, Niinemets Ü, Oleksyn J, *et al.* 2005. Modulation of leaf economic traits and trait relationships by climate. *Global ecology and biogeography: a journal of macroecology* **14**: 411–421.
